# Instrumental variable analysis with categorical treatment

**DOI:** 10.1177/09622802241281960

**Published:** 2024-10-30

**Authors:** Amir Aamodt Kazemi, Inge Christoffer Olsen

**Affiliations:** 1 Department of Research Support for Clinical Trials, 155272 Oslo University Hospital, Oslo, Norway; 2 Oslo Centre for Biostatistics and Epidemiology, Institute of Basic Medical Sciences, Faculty of Medicine, University of Oslo, Oslo, Norway

**Keywords:** Causal inference, choice theory, principal stratification, rheumatoid arthritis, tumor necrosis factor inhibitor

## Abstract

Current instrumental variable methodology focuses mainly on estimating causal effects for a dichotomous or an ordinal treatment variable. Situations with more than two unordered treatments are less explored. The challenge is that assumptions needed to derive point-estimators become increasingly stronger with the number of relevant treatment alternatives. In this article, we aim at deriving causal point-estimators for head-to-head comparisons of the effect of multiple relevant treatments or interventions. We will achieve this with a set of plausible and well-defined rationality assumptions while only considering ordinal instruments. We demonstrate that our methodology provides asymptotically unbiased estimators in the presence of unobserved confounding effects in a simulation study. We then apply the method to compare the effectiveness of five anti-inflammatory drugs in the treatment of rheumatoid arthritis. For this, we use a clinical data set from an observational study in Norway, where price is the primary determinant of the preferred drug and can therefore be considered as an instrument. The developed methodology provides an important addition to the toolbox for causal inference when comparing more than two interventions influenced by an instrumental variable.

## 1. Introduction

The ability to estimate causal effects, not only associations or predictions, is of utmost importance in medical research. The simplest method to estimate causal effects of an intervention is randomized controlled trials (RCTs). In an ideal RCT, the randomization breaks all alternative causal pathways between the treatment and the outcome. The residual variance can then, under some limitations, be assumed to be random. However, in some cases, RCTs are expensive or not feasible. The alternative is to use observational data. The challenge is that standard statistical analyses on observational data require strong assumptions to provide valid causal effect estimates, including that of no unobserved confounding effects. When these assumptions are violated, the results can be biased.

Instrumental variables (IV) analysis, since its first application by Wright,^1^ has been a popular choice of analysis method in agriculture, economy and social sciences to estimate causal effects from observational data in the presence of unobserved confounding. The main idea is to take advantage of an exogenous variable that affects the choice of treatment or intervention, but is otherwise independent from the causal structure of the system. This is called an *instrumental variable* (IV).^2^

### 1.1. Literature review

Application of IV analysis in medicine and clinical research has been, until recent decades, somewhat limited. Imbens^3^ argued that this might have been due to the early literature on IV being written with economic questions in mind, such as the effect of interventions in markets. Therefore, the authors used theoretical economic language, such as supply and demand, which may have appeared difficult to translate to other fields. Another reason might be the extended use of RCTs to identify causal effects in clinical research compared to other fields such as econometrics. A literature review by Cawley^4^ showed that the application of this method in the medical field has increased significantly in the recent decades (after 1990). The wave of methodological publications related to IV analysis in the early 1990s, such as Angrist and Imbens^5,6^, might have contributed to this fact.

The vast majority of the IV literature considers the situation of a dichotomous treatment variable. Situations with more than two unordered treatments are much less explored in the IV literature in general and not at all in the medical literature to our knowledge. The challenge is that assumptions needed to enable effect identification become increasingly stronger with the number of possible treatments. For instance, Swanson et al.^7^ argued that performing IV analysis to compare two treatments, when filtering out other relevant alternatives in a naive way, is likely to yield biased estimators.

To derive causal point-estimators for the treatment effect in the dichotomous case, current methodology requires assuming either *homogeneity* or *monotonicity*.^5^ In the case of a nominal treatment variable with more than two possible values, we will show that homogeneity alone is not enough to derive causal estimators. Generalizing monotonicity to a set of assumptions for a nominal multi-valued treatment, that are both plausible and enable effect estimation, has proven to be a rather difficult task.^8,9^

In the mission to generalize IV analysis, Frangakis and Rubin^10^ achieved a breakthrough by developing a framework for *principal stratification*, encompassing the conventional IV methods. Principal stratification provided a fresh perspective on the assumptions needed for causal effect estimation. Inspired by this framework, a number of papers, such as Cheng and Small^11^, Kirkeboen et al.^12^, Hull^13^, and Blackwell^14^, were published developing and applying IV analysis in cases with polytomous treatment variables, deriving causally interpretable estimators under some clearly stated additional assumptions. However, these attempts made strong assumptions on the existence of certain principal strata and in some cases, limited the number of possible treatment alternatives to three, restricting the applicability of these results. Recently, Heckman and Pinto^15^ developed and presented an IV framework without any limitations on the maximum possible number of treatment alternatives or ordinality. They proposed to use axioms of choice theory to limit the number of possible principal strata. They then went on to suggest an *unordered monotonicity* assumption, where there can only exist changes in the instrument causing one-way flows either in or out of each treatment alternative. Monotonicity in the dichotomous case is a special case of unordered monotonicity. Given the study design, it’s simple to argue whether or not this assumption holds. When the unordered monotonicity assumption is fulfilled, they were able to derive causally interpretable effect estimators. Even though Heckman and Pinto^15^ provided exceptional insight into the required assumptions for identification of causal effects with an IV approach and presented a clear link between this framework and choice theory, their assumption of unordered monotonicity has been criticized in the literature, for instance by Lee and Salanié,^16^ for not being realistic in many use-cases. As we discuss in Supplemental Appendix Subsection B.5, it’s actually plausible to think that this assumption is violated in the clinical use-case presented in Subsection 1.3 of this article.

### 1.2. Statistical motivation

In this article, we will present a causal framework for head-to-head comparisons of multiple treatments or interventions, avoiding the assumption of unordered monotonicity. We will achieve this by only considering ordinal instruments. This means that the instrument can be formulated as a ranking of the treatment alternatives where a lower rank means higher preference. Our approach is to a large degree inspired by the efforts made by Heckman and Pinto.^15^ Similar to their solution, we also take advantage of the properties of binary matrices. However, we will replace their unordered monotonicity with our own set of assumptions, which we argue are more applicable in clinical use-cases similar to ours.

The aim is to develop an IV framework for situations with several unordered treatments. We will do our best to avoid economic language that has the potential to create confusion among epidemiologists and clinical researchers, while borrowing some notation from econometrics that helps us transform clinical questions into statistical ones. This approach provides a natural gateway for clinical assumptions to enter and contribute to the analysis. In addition, it provides a framework where one doesn’t need to make a judgment about the existence of every single principal stratum, as Heckman and Pinto^15^ did in the examples they solved with choice theory, not assuming unordered monotonicity.

### 1.3. Clinical motivation

The framework presented here has been motivated by the need for head-to-head comparisons of the effectiveness of biologic drugs in the treatment of patients with inflammatory joint diseases. To our knowledge, this is the first application of any IV method to assess the effectiveness of these medications using observational data.

Tumor necrosis factor inhibitors (TNFis) are biologic drugs used to decrease and hopefully stop inflammation in patients with inflammatory arthritis diseases. We have looked at five TNFis approved to be used in Norway, namely infliximab, golimumab, certolizumab pegol, etanercept, and adalimumab.

Rheumatoid arthritis (RA)^17^ is an inflammatory disease affecting the musculoskeletal system associated with significant morbidity and increased mortality.^18^ The treatment of RA has been revolutionized by the introduction of biologic treatments and particularly TNFis. Several structurally different TNFis have emerged on the market in the last 20 years, and many have been proven effective in the treatment of RA.^19^ However, head-to-head comparison studies of different TNFis within approved indications are scarce. At the introduction of TNFis there were concerns regarding both long-term safety of the drugs and generalizability of the results from RCTs due to selective patient inclusion.^20^ For this reason, large observational studies of patients initiating biologic treatments were introduced in several European countries, including Norway. The Norwegian disease-modifying anti-rheumatic drug (NOR-DMARD) study has registered treatments and outcomes of biologic treatments for 20 years, giving a comprehensive database for real-world evidence. This data set will be used in our analysis for measuring the effectiveness of the aforementioned TNFis in the treatment of RA.

Treatment with TNFis has come at a significant cost to the payer, that is, the public health service in Norway. The Norwegian Drug Procurement Cooperation (NDPC) was established in 1995 and manages a tender system where the health regions collaborate to procure favorable agreements for hospital administered treatments. The tender system works such that every year, the pharmaceutical companies have been invited to present a price for their biologic treatment to a committee consisting of clinical representatives from all Norwegian health regions. The committee then reviews the prices and the biologic treatment with the lowest price has been selected to be used as first line treatment for all health regions. Although the bidding price presented by the companies are not public information, we, as well as most rheumatologists in Norway have had access to all bidding prices from 2010 to 2019. Clinicians are required to adhere to the NDPC’s recommended treatment unless there are strong medical reasons not to. In this setting, price is the main factor determining the preferred drug, based on the widespread, but largely unproven assumption that TNFis are equally effective on a group level. This article is motivated by the prospect of using the tender system and the price ranking of biologic treatments as an IV. Together with clinical data from the NOR-DMARD registry, the combination can provide the data basis for estimating causally interpretable treatment effects for the TNFis of interest.

### 1.4. Outline

Motivated by the methodological challenges and the clinical question introduced in this section, we first lay out the notation and assumptions needed for causal inference with IV analysis in Section 2. In Subsection 2.2.1, we illustrate why dichotomous IV assumptions are difficult to generalize to a setting with polytomous treatments. Then we go on to introduce our alternative notation and assumptions to enable effect estimation in those settings.

In Section 3, we use the assumptions introduced in Section 2 to derive estimators of the causal treatment effects and provide tools to interpret the estimates.

Finally, in Section 5, we will present the results of applying the methods developed in Section 3 to real-world data in order to answer the clinical question posed in Subsection 1.3. Clinical discussion of these results is out of the scope of this work. The applicability of the method developed in this article to answer our clinical question will, however, be further discussed in Section 6.

In the Appendix, we provide all mathematical details in addition to further clinical and methodological discussions and examples. In Section 4, we present a simulation study with randomly generated data in a simple setting with three relevant treatment alternatives. We then compare the performance of the estimator derived in Section 3 with the dichotomous IV and a naive estimator.

## 2. Notation and assumptions

To discuss the problem at hand in a mathematical language, we need to define some basic terms and notation.

Definition 1 **The decision team** is defined as a combination of the patient, the physician and other health personal, whose preferences and status affects the choice of treatment. In the econometrics literature, the word *agent* is used to refer to this entity. We represent the decision team by the letter w .

### 2.1. Basic notation

Let T be the treatment variable and Z be a vector of the treatment alternatives sorted by the level of encouragement in descending order. Let V be the set of all observed and unobserved confounding factors affecting the choice of treatment and the outcome. Let Y be the outcome.

Definition 2 A treatment alternative, t , is **relevant** for a decision team if and only if the probability of that decision team choosing t is greater than zero.

Namely: P(T=t|V=v)>0 . A treatment alternative, t , is relevant for a population if and only if t is relevant for some decision teams in that population.

Definition 3 **The counterfactual outcome** of treatment t for decision team w , Y(t) , is defined as the outcome, had w been forced to take treatment t by an intervention.

Definition 4 **The average treatment outcome (ATO)** of t , E(Y(t)) , is defined as the expected counterfactual outcome under T=t in the whole population.

Definition 5 **The average treatment effect (ATE)** of t compared to $t^{\prime }$ is defined as $E(Y(t)-Y(t^{\prime }))$ .

Note that, as Pearl^2^ argues, the ATO is different from E(Y|T=t) which is the expected outcome for those who actually chose treatment t .

Definition 6 **The local ATO (LATO)** and **the local ATE (LATE)** for a sub-population Σ , are defined as the ATO and ATE in that sub-population, respectively. Namely:

$$\begin{array}{rl} & \text{LATO: }E(Y(t)|w\in \Sigma )\\ & \text{LATE: }E(Y(t)-Y(t^{\prime })|w\in \Sigma ) \end{array}$$

For a head-to-head comparison of two treatment alternatives, t and $t^{\prime }$ , in a sub-population Σ , we need to estimate the LATE of t compared to $t^{\prime }$ in that sub-population. In this article, we will achieve this by estimating the LATO of t and $t^{\prime }$ in Σ and then subtracting one from the other.

The following equation, which is known as *consistency*,^2^ governs the relationship between the observed and the counterfactual outcomes:

(1) $$Y=\sum_{t}Y(t)\cdot 1[T=t]$$

where Y is the observed outcome, T is the observed treatment, and 1[⋅] is the indicator function.

In this article, we will also discuss the dichotomous IV method as a special case of the framework developed here. In the dichotomous case with a treatment ( a ) and a control ( b ), we would have: $z_{a}=(a,b)\;\&\;z_{b}=(b,a)$ . Then $z_{a}$ represents all the values of the instrument that encourage taking the treatment (a) , and $z_{b}$ represents all the values for the instrument that discourage taking the treatment (encouraging the control (b) ).

### 2.2. Assumptions

To estimate causal effects with our framework, we need the main three IV assumptions.

Assumption The main IV assumptions can be expressed as follows:

(i)
$T=f_{T}(Z,V,\delta )$ : The choice of treatment only depends on the instrument, the confounding effects and a random residual δ .
(ii)
$Y=f_{Y}(T,V,\epsilon )$ : The outcome only depends on the choice of treatment, the confounding effects and a random residual ϵ .
(iii)
V,Z,δ , and ϵ are mutually independent.

The most important implication of these assumptions is that Z does not have any effect on Y other than through its effect on T . In Supplemental Appendix Subsection B.1, we provide an explanation of what these assumptions mean in our clinical use-case.

#### 2.2.1. Homogeneity and monotonicity

In this subsection, we will illustrate why Assumptions (i) to (iii) together with one of two other assumptions denoted homogeneity and monotonicity are enough for identification of causal effects in the dichotomous case but not in the case of >2 nominal treatment alternatives.

In the dichotomous case, Angrist and Imbens^5^ divided the population of decision teams into four groups depending on how decision teams respond to a change in the instrument. The four groups are always-takers of a ( $AT_{a}$ ), never-takers of a ( $NT_{a}$ ), compliers ( C ), and defiers ( D ). Note that never-takers of a are always-takers of b . Frangakis and Rubin^10^ considered these groups as principal strata, denoted by S . Note that S is independent of Z .

Definition 7 Homogeneity states that Y(a)−Y(b) is independent from the confounding effects, and consequently also from the principal stratum.

Definition 8 Monotonicity states that decision teams can only respond positively or stay neutral to a change in the instrument that encourages T=a . In other words, there exists no defiers in the population ( P(D)=0 ). Notationally: $1[T(z_{a})=a]\geq 1[T(z_{b})=a]$ .

Monotonicity is basically a rationality assumption. When price is the instrument, as in our clinical case, monotonicity states that there are no decision teams that would choose treatment a if it is expensive and avoid it if it is cheap. Going forward in this article, we will need to generalize this rationality assumption to incorporate a categorical treatment variable.

Proposition 1 If only the two treatment alternatives a and b are relevant for a population ( P(T=a)+P(T=b)=1 ), given Assumptions (i) to (iii) and homogeneity, the ATE can be expressed as follows:

(2) $$E(Y(a)-Y(b))=\frac{E(Y|Z=z_{a})-E(Y|Z=z_{b})}{P(T=a|Z=z_{a})-P(T=a|Z=z_{b})}$$

Replacing homogeneity with monotonicity, the right hand-side of equation (2) is equal to the LATE for the compliers. Namely:

(3) $$\begin{array}{r} E(Y(a)-Y(b)|S=C)=\frac{E(Y|Z=z_{a})-E(Y|Z=z_{b})}{P(T=a|Z=z_{a})-P(T=a|Z=z_{b})} \end{array}$$

**Proof.** Supplemental Appendix Subsection A.1.

Every quantity on the right-hand side of equation (2) is possible to estimate. The ATE (LATE) is, therefore, identifiable given homogeneity (monotonicity). Proposition 1 is the conventional dichotomous IV estimator.

In most cases, it can be argued that homogeneity is an implausible assumption. Monotonicity was introduced by Angrist and Imbens,^5^ as a replacement for homogeneity. In the subsequent IV literature, monotonicity is central for derivation of estimators for the LATE, which estimate the treatment effect only for those whose choice of treatment is affected by the value of the instrument.

We will illustrate why equation (2) does not generally hold if there are three or more relevant unordered treatment alternatives. For simplicity, we look at the case with three treatment alternatives a , b , and c , and assume homogeneity and monotonicity. Generalizing monotonicity from a dichotomous to a polytomous nominal treatment is not a straight forward task. For the sake of this argument, let us define *simple monotonicity* as the natural expansion of monotonicity in the dichotomous case.

Definition 9 **Simple monotonicity** states that if a decision team chooses T=t under $Z\neq z_{t}$ , then that decision team would necessarily also choose T=t under $Z=z_{t}$ . Notationally:

$$1[T(z_{t})=t]\geq 1[T(z_{t^{\prime }})=t]\;\forall \;t\neq t^{\prime }$$

where $T(z_{t})$ is the counterfactual choice of treatment under a value of the instrument, $z_{t}$ , which encourages T=t above all other alternatives.

In the dichotomous case, Angrist and Imbens divide the population of decision teams into four groups depending on how decision teams respond to a change in the instrument. These groups are summarized in Tables 1. Note that never-takers of a are always-takers of *b*. Frangakis and Rubin consider these groups as principal strata, denoted by *S*. Note that *S* is independent of *Z*.

**Table 1.** Summary of counterfactual choices of treatment in all principal strata with two treatment alternatives.

Principal strata ( S )	$T|Z=z_{a}$	$T|Z=z_{b}$
Always-takers of *a* ( $AT_{a}$ )	*a*	*a*
Never-takers of *a* ( $NT_{a}$ )	*b*	*b*
Compliers ( C )	*a*	*b*
Defiers ( D )	*b*	*a*

**Table 2.** Summary of counterfactual choices of treatment alternatives in all principal strata under simple monotonicity with three treatment alternatives.

Principal strata	$T|Z=z_{a}$	$T|Z=z_{b}$	$T|Z=z_{c}$
Always-takers of *a* ( $AT_{a}$ )	*a*	*a*	*a*
Always-takers of *b* ( $AT_{b}$ )	*b*	*b*	*b*
Always-takers of *c* ( $AT_{c}$ )	*c*	*c*	*c*
Never-takers of *a* ( $NT_{a}^{b}$ )	*b*	*b*	*c*
Never-takers of *a* ( $NT_{a}^{c}$ )	*c*	*b*	*c*
Never-takers of *b* ( $NT_{b}^{a}$ )	*a*	*a*	*c*
Never-takers of *b* ( $NT_{b}^{c}$ )	*a*	*c*	*c*
Never-takers of *c* ( $NT_{c}^{a}$ )	*a*	*b*	*a*
Never-takers of *c* ( $NT_{c}^{b}$ )	*a*	*b*	*b*
Compliers ( C )	*a*	*b*	*c*

Remark 1 Given homogeneity and simple monotonicity for three relevant treatment alternatives, equation (2) does not generally hold. Namely:

$$E(Y(a)-Y(b))\neq \frac{E(Y|Z=z_{a})-E(Y|Z=z_{b})}{P(T=a|Z=z_{a})-P(T=a|Z=z_{b})}$$

**Proof.** Supplemental Appendix Subsection A.2.

With more than three alternatives the problem only exacerbates, as the number of principal strata grows rapidly in the absence of stricter monotonicity assumptions, resulting in no identification. One might consider constructing estimators based on other simple generalizations of equation (2), for example, one that filters out all observations with T=c and performs dichotomous IV analysis to compare a and b . It can be shown that this estimator is also generally inconsistent.^7^

To gain intuition about the problem at hand, it’s worth noting that a nominal treatment variable with more than two levels gives rise to ambiguities which are hardly solvable by assuming homogeneity or straightforward generalizations of monotonicity. In the dichotomous case with the only possible alternatives for treatment being a and b , the counterfactual treatment choice given T≠a is necessarily T=b . This means the answer to the question “what treatment had the patient received if not a ?” is b . This question, however, does not have a unique answer if there exist more than two relevant treatment alternatives. Therefore, as illustrated in Table 2, to achieve consistency, the number of principal strata must be increased to a point where identification is impossible. Generalizations of the monotonicity assumption can then be used to limit the number of principal strata. However, as illustrated earlier, simple monotonicity is not strong enough to enable identification. On the other hand, it’s challenging to construct a stronger monotonicity assumption that enables effect identification while still being plausible. To achieve this, we will take advantage of our instrument’s ordinality.

#### 2.2.2. Introduction to our framework

We have illustrated that the dichotomous IV methodology doesn’t generalize well to use-cases like ours. Here, an attempt is made to present a framework that can provide estimates for the LATE in the presence of more than two relevant treatment alternatives. We will avoid assuming homogeneity, as this is an unrealistic assumption in most use-cases. To achieve identifiablity, we essentially need to dismiss some of the principal strata with additional assumptions. Researchers could achieve this goal by going through all principal strata and dismiss some of them based on field expertise, the data generating process or common sense. This would be in style with the choice theoretic methods presented by Heckman and Pinto.^15^ However, we aim at providing plausible general assumptions that dismiss principal strata systematically, ultimately leading to identifiable point-estimators without the need of a field expert having to make an assumption on the existence of every single stratum.

An interpretation of our methodology is that we utilize revealed preferences to extract information about the confounding effects. What is a revealed preference? If a decision team chooses a more expensive treatment alternative, we at least know that for some reason, they had a preference for avoiding the cheaper treatment. We also know that they didn’t have a preference for avoiding the treatment that they actually chose. This information is a revealed preference and will be used in the derivation of our estimators.

#### 2.2.3. Additional notation and assumptions

Our proposed method will be introduced through the following notation and assumptions.

Definition 10 **Adherence set** is defined as the set of all treatment alternatives that are relevant for a decision team. Denote adherence set with A . In our notation, A cannot be an empty set. If *no-treatment* is an alternative, it must be included as one of the possible treatment alternatives, such that A never is an empty set. Notationally: A={t|P(T=t|V,δ)>0} .

Let $\Omega_{a}$ be the set of all decision teams for which the adherence set is a : $\Omega_{a}=\{w|A=a\}.$

Note that even though the adherence set is not entirely observable, we can still extract some information about it from the data leveraging revealed preferences. Further discussions and examples of this variable in the context of our clinical use-case are provided in Supplemental Appendix Subsection B.2.

Assumption Our additional assumptions are formalized as the following:

(iv)
$A=f_{A}(V,\delta )$ : The adherence set of a decision team only depends on the confounding effects and a random residual. Note from Assumption (i), δ⊥⊥V,Z,ϵ .
(v)
$\Omega_{a}\cap \Omega_{a^{\prime }}=\emptyset$ : There is no decision team that has two different adherence sets.
(vi)
T=g(Z,A) : The choice of treatment is only a function of the adherence set and the instrument. In addition, g must be known.

**Figure 1.**
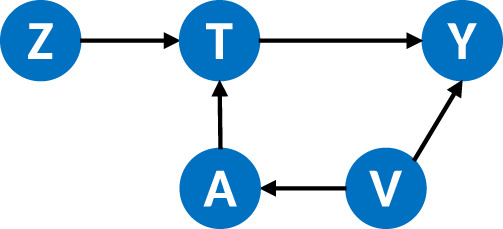
The directed acyclic graph defined by Assumptions (i) to (vi).

Given Assumptions (i) to (vi), the directed acyclic graph (DAG) in Figure 1 illustrates the causal structure of the system we are presenting. Given these assumptions adherence sets can be considered equivalent to principal strata, as defined by Frangakis and Rubin.^10^

The function g in Assumption (vi) is where the choices made by the decision teams can be modeled. As long as this function is known and does not contradict any of the other assumptions, the analysis can continue on to assessing identifiablity of the LATEs. However, in this article, we are particularly interested in the following choice function:

(4) $$g(Z,A)=t_{\min(\{i|t_{i}\in A\})},\;\text{where }Z=(t_{1},t_{2},\ldots ,t_{N_{T}})$$

This function selects the lowest ranking treatment in Z , that is, in A . In our clinical use-case, g takes in the price-ordered list of all treatments and the adherence set. For each decision team, it then selects the cheapest alternative in the adherence set of that decision team. Going forward, in this article, we will always assume that the choices made by the decision teams are governed by equation (4). Further discussion of this choice model is provided in Supplemental Appendix Subsection B.3.

#### 2.2.4. Alternative assumptions

Heckman and Pinto^15^ set up an alternative set of assumptions denoted as *unordered monotonicity* to identify causal effects in a similar setting to ours. In Supplemental Appendix Subsection B.5, we compare our set of assumptions to unordered monotonicity.

## 3. Identification and estimation

In this section, we derive estimators for the LATE in a sub-population, Σ , given Assumptions (i) to (vi) and the choice model defined in equation (4). In the dichotomous case, Σ is the usual set of compliers, namely $\Sigma =\Omega_{\left\{a,b\right\}}$ . In the case of a multi-valued treatment variable, the definition of Σ is slightly more complex, but still interpretable. In Subsection 3.5, we will provide tools to interpret this sub-population.

### 3.1. Independence conditions (ICs) and standardization

The following ICs follow from Assumptions (i) to (vi):

1.
Y(t)⊥⊥T|A

2.
A⊥⊥Z

3.
Y⊥⊥Z|(A,T)

Lemma 1 Following equation (1) and IC 1, we have:

$$E(Y(t)|A=a)=E(Y|A=a,T=t)\;\forall \;a\in \{a^{\prime }\;|\;t\in a^{\prime }\}$$

**Proof.** Supplemental Appendix Subsection A.3.

Expressed in a causal language, Lemma 1 states that given the adherence set, the counterfactual outcome is equal to the observed outcome since conditioning on the adherence set breaks all backdoor causal pathways between the treatment and the outcome.

Lemma 2 For any treatment, t , and any subset of the adherence sets, Π⊆{a|t∈a} , the LATO in the corresponding sub-population, Σ={w|A∈Π} , can be written as follows:

(5) $$\begin{array}{r} E(Y(t)|w\in \Sigma )=\frac{1}{P(A\in \Pi )}\sum_{a\in \Pi }E(Y|A=a,T=t)P(A=a) \end{array}$$

**Proof.** Supplemental Appendix Subsection A.4.

By this, we have shown that if the adherence set of every decision team in the population was known, we would have been able to estimate the LATO of t for any population of decision teams for whom t was relevant. Unfortunately, the adherence set is not observable!

### 3.2. Utilizing observables

What can we observe in the data? We observe the instrument, Z , the treatment T and the outcome Y . Under each available value for Z , we can estimate the probability of choosing any treatment, characterized as

P(T=t|Z=z) . In addition, we can estimate

E(Y|T=t,Z=z) . The product of these two quantities is important in our derivations, namely

E(Y|T=t,Z=z)P(T=t|Z=z) . An interpretation of this product would be t ’s contribution to the expected outcome under Z=z .

We will connect these observed quantities with the quantities that we want to estimate, namely

E(Y|A=a,T=t) and P(A=a) . If we can manage to estimate these, we can plug in the estimated values in equation (5), and we will have an estimate for the LATOs.

Proposition 2 Given ICs 1–3, we have:

(6) $$\begin{array}{rl} & P(T=t|Z=z)=\sum_{a}1[T=t|A=a,Z=z]P(A=a) \end{array}$$

(7) $$\begin{array}{rl} & E(Y|Z=z,T=t)P(T=t|Z=z)=\sum_{a}1[T=t|A=a,Z=z]E(Y|A=a,T=t)P(A=a) \end{array}$$

**Proof.** Supplemental Appendix Subsection A.5.  □

The left hand-sides of equations (6) and (7) are observable, while the right hand-sides consist of the quantities of interest. Note that the simplicity of these equations stems from the fact that the choice function defined in equation (4) and therefore the indicator function 1[T=t|A=a,Z=z] are deterministic. We have connected the observables and the unknowns in a system of equations. The “only” remaining task is to solve this system.

### 3.3. Vectorization and matrix notation

To solve the system of equations represented by equations (6) and (7), we reformulate these equations into matrix forms. This can be done in a similar manner as it was done by Heckman and Pinto.^15^ Here we borrow their notation.

Definition 11 Formulate the observables and the unknowns as the following vectors:

$$\begin{array}{rl} P_{Z}(t) & =[P(T=t|Z=z_{1}),\ldots ,P(T=t|Z=z_{N_{Z}}){]}^{T}\\ P_{A} & =[P(A=a_{1}),\ldots ,P(A=a_{N_{A}}){]}^{T}\\ Q_{Z}(t) & =[E(Y|Z=z_{1},T=t),\ldots ,E(Y|Z=z_{N_{Z}},T=t){]}^{T}⊙P_{Z}(t)\\ Q_{A}(t) & =[E(Y|A=a_{1},T=t),\ldots ,E(Y|A=a_{N_{A}},T=t){]}^{T}⊙P_{A} \end{array}$$

where ⊙ is the Hadamard product.

Definition 12 For any treatment, t , let $B_{t}$ denote a binary matrix of the dimension $N_{Z}\times N_{A}$ . Each row of $B_{t}$ corresponds to a value for the instrument and each column corresponds to an adherence set. The elements in $B_{t}$ take value 1 if the decision teams in the corresponding adherence set choose to take treatment t under the corresponding value of the instrument. Notationally, we define an element in the *i*-th row and *n*-th column of matrix $B_{t}$ by: $B_{t}[i,n]=1[T=t|Z=z_{i},A=a_{n}]$ .

Note that given the choice function defined in equation (4) and the observations of Z , the $B_{t}$ s are known.

Lemma 3 Equations (6) and (7) can be reformulated into a matrix form:

(8) $$\begin{array}{rl} Q_{Z}(t) & =B_{t}Q_{A}(t) \end{array}$$

(9) $$\begin{array}{rl} P_{Z}(t) & =B_{t}P_{A} \end{array}$$

If $B_{t}$ is invertible, equation (8) can be solved and then the estimated value for $Q_{A}(t)$ can be plugged into equation (5) to identify the LATO of t for any combination of adherence sets. However, invertiblity of $B_{t}$ is an extremely strong requirement, which would not be satisfied in most use-cases.

### 3.4. Pseudo-inversion and identification

When $B_{t}$ is not invertible, some LATOs might still be identifiable. To achieve this goal, still similar to the derivations done by Heckman and Pinto,^15^ we take advantage of the Moore–Penrose pseudo-inversion.

Definition 13 Let $B_{t}^{+}$ be the pseudo-inverse of $B_{t}$ and define as follows:

$$K_{t}=I_{N_{A}}-B_{t}^{+}B_{t}$$

where $I_{N_{A}}$ is the identity matrix of dimensions $N_{A}\times N_{A}$ . Let $b_{t}$ be a non-zero binary vector of size $N_{A}$ , such that:

(10) $$b_{t}K_{t}=0$$

The elements of $b_{t}$ correspond naturally to the adherence sets. Denote the element of $b_{t}$ that corresponds to the adherence set a as $b_{t}[a]$ .

Definition 14 For each $b_{t}$ , consider the following subset of all adherence sets and sub-population of decision teams:

$$\begin{array}{rl} \Pi (b_{t}) & =\{a|b_{t}[a]=1\}\\ \Sigma (b_{t}) & =\{w|A\in \Pi (b_{t})\} \end{array}$$

where A is the adherence set of decision team w .

Theorem 1 If for two different treatments t and $t^{\prime }$ , there exists a vector b , such that:

$$bK_{t}=bK_{t^{\prime }}=0$$

then $bQ_{A}(t)$ , $bQ_{A}(t^{\prime })$ , and $bP_{A}$ can be identified as follows:

(11) $$\begin{array}{rl} bQ_{A}(\tau ) & =bB_{\tau }^{+}Q_{Z}(\tau )\;\forall \;\tau \in \{t,t^{\prime }\} \end{array}$$

(12) $$\begin{array}{rl} bP_{A} & =bB_{t}^{+}P_{Z}(t)=bB_{t^{\prime }}^{+}P_{Z}(t^{\prime }) \end{array}$$

Consequently, the LATEs for sub-population Σ(b) can be calculated as follows:

(13) $$E(Y(t)-Y(t^{\prime })|w\in \Sigma (b))=\frac{bB_{t}^{+}Q_{Z}(t)}{bB_{t}^{+}P_{Z}(t)}-\frac{bB_{t^{\prime }}^{+}Q_{Z}(t^{\prime })}{bB_{t^{\prime }}^{+}P_{Z}(t^{\prime })}$$

**Proof.** Supplemental Appendix Subsection A.6.

In Supplemental Appendix Subsection B.4, further discussion of this identifiablity result is provided together with some hypothetical examples of its application.

#### 3.4.1. The plug-in estimator

Given a finite sample, $P_{Z}$ and $Q_{Z}$ can be estimated from the data. Denote the estimates as ${\hat{P}}_{Z}$ and ${\hat{Q}}_{Z}$ .

Definition 15 **The plug-in categorical IV (CIV)** estimator for the LATEs can be constructed as follows:

(14) $${\hat{E}}_{CIV}(Y(t)-Y(t^{\prime })|w\in \Sigma (b))=\frac{bB_{t}^{+}{\hat{Q}}_{Z}(t)}{bB_{t}^{+}{\hat{P}}_{Z}(t)}-\frac{bB_{t^{\prime }}^{+}{\hat{Q}}_{Z}(t^{\prime })}{bB_{t^{\prime }}^{+}{\hat{P}}_{Z}(t^{\prime })}$$

Corollary 1 By applying the Taylor expansion, assuming that the number of observations is homogeneous over the values of the instrument and that ${\hat{P}}_{Z}$ and ${\hat{Q}}_{Z}$ are unbiased:

$$\begin{array}{r} E({\hat{E}}_{CIV}(Y(t)-Y(t^{\prime })|w\in \Sigma (b)))=E(Y(t)-Y(t^{\prime })|w\in \Sigma (b))+O(n^{-1}) \end{array}$$

where n is the number of observations in the data set. In other words, the plug-in CIV estimator is asymptotically unbiased.

**Proof.** Supplemental Appendix Subsection A.7.

### 3.5. Describing sub-populations

The sub-population, Σ(b) , can be described in terms of adherence. For instance, in a setting with three alternatives, Σ(b)={w|A={a,b}} , is the set of decision teams that comply to the instrument with respect to a and b but never choose c . However, if some covariates are observed, this population can also be described in terms of those covariates. Assume some covariate, X , is observed.

Definition 16 The observable and the unknown expectations of X are denoted as follows:

$$\begin{array}{rl} L_{Z}(t) & =[E(X|Z=z_{1},T=t),\ldots ,E(X|Z=z_{N_{Z}},T=t){]}^{T}⊙P_{Z}(t)\\ L_{A} & =[E(X|A=a_{1}),\ldots ,E(X|A=a_{N_{A}}){]}^{T}⊙P_{A} \end{array}$$

Proposition 3 Assuming X⊥⊥Z and X⊥⊥T|A , we have:

(15) $$E(X|w\in \Sigma (b))=\frac{bB_{t}^{+}L_{Z}(t)}{bB_{t}^{+}P_{Z}(t)}=\frac{bB_{t^{\prime }}^{+}L_{Z}(t^{\prime })}{bB_{t^{\prime }}^{+}P_{Z}(t^{\prime })}$$

**Proof.** Follow a similar argument to the proof of Theorem 1, presented in Supplemental Appendix Subsection A.6.

This allows us to show adjusted or balanced descriptions of covariates for each treatment in the sub-population Σ(b) , much alike baseline tables in regular reports of RCTs.

### 3.6. Conditional instruments

**Figure 2.**
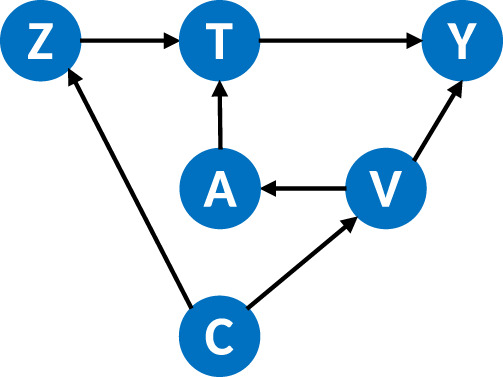
An example directed acyclic graph (DAG) for a conditional instrument.

The exogeneity of the instrument is the cornerstone of the IV method. Truly exogenous instruments are hard to come by and one could always argue in favor of one or another backdoor causal pathway between the proposed instrument and the outcome. Figure 2 provides an example of a such situation, where Z is only an exogenous instrument conditional on C . It’s necessary that the methodology provides tools to block identified potential backdoor causal pathways. The method presented in this article can easily be generalized to incorporate conditional instruments. This is provided in Supplemental Appendix Subsection B.6.

### 3.7. Testable implications of the assumptions

While equations (11) and (12) are used to identify LATOs, equations (12) and (15) actually have some testable implications. Notice that t is missing from the left hand-side of these equations. This means that for every triplet $\left(b,t,t^{\prime }\right)$ , where $t\neq t^{\prime }$ and equation (10) holds for both t and $t^{\prime }$ , we must have

$$bB_{t}^{+}P_{Z}(t)=bB_{t^{\prime }}^{+}P_{Z}(t^{\prime })$$

If a covariate, X , is observed, we also must have

$$bB_{t}^{+}L_{Z}(t)=bB_{t^{\prime }}^{+}L_{Z}(t^{\prime })$$

These results can be used to develop tests for the assumptions underlying the model. Further interpretation of these results is given in Supplemental Appendix Subsection B.7.

## 4. Simulation study

To further build trust in the methods presented in Section 3, we present the results of a simulation study, built up as follows:

1. Present the simulation mechanism.
2. Simulate from the simulation mechanism including unobserved confounding variables affecting both treatment choice and outcome.
3. Compute the treatment effect estimates using our proposed method, and compare to estimates based on other estimators.

### 4.1. Simulation mechanism

We consider the simplest case of three relevant treatment alternatives, which are represented here as $t_{1-3}$ . To achieve identifiablity, we consider the following values for the instrument:

$$\begin{array}{rl} z_{1} & =[t_{1},t_{2},t_{3}]\;z_{2}=[t_{2},t_{1},t_{3}]\\ z_{3} & =[t_{2},t_{3},t_{1}]\;z_{4}=[t_{3},t_{2},t_{1}] \end{array}$$

Decision teams are assigned a value for the instrument at random, with equal probability of each value. Namely $P(Z=z_{j})=0.25\;\forall \;j\in \{1,2,3,4\}$ .

Two independent confounding effects, $V_{1}$ and $V_{2}$ are generated as binary variables such that $V_{1},V_{2}∼B(n,0.5)$ . These variables will affect the probability of treatments and outcome. They will, however, be removed from the simulated data set, as they represent unobserved confounding effects.

Given the instrument and the confounding effects for each decision team, the treatment is then chosen randomly with the following probabilities for $t_{1-3}$ :

$$\begin{array}{r} P(T=t_{i}|V_{1}=v_{1},V_{2}=v_{2},Z=z_{j})\propto e^{3-k}+v_{1}\cdot e^{3}\cdot 1[i=1]+v_{2}\cdot e^{3}\cdot 1[i=2]\text{ where }z_{j}=(t_{1}^{\prime },t_{2}^{\prime },t_{3}^{\prime })\text{ and }t_{k}^{\prime }=t_{i} \end{array}$$

For each $t_{i}$ , given Z , $V_{1}$ , and $V_{2}$ , we find the place of $t_{i}$ in Z . Let’s say that $t_{i}$ is the k -th cheapest alternative. We then set the probability of $t_{i}$ proportional to $e^{3-k}$ , such that the probability of a treatment decreases exponentially with its price. If $V_{1}=1$ , we add $e^{3}\approx 20$ to the probability of $t_{1}$ , and if $V_{2}=1$ , we add $e^{3}$ to the probability of $t_{2}$ . We then normalize such that the probabilities sum to one. As illustrated in Supplemental Appendix Subsection B.11, this mechanism produces a similar probability distribution over the treatment alternatives to the one observed in our clinical use-case.

Note that this simple choice mechanism actually violates Assumption (vi), because in this choice mechanism, all decision teams have a non-zero probability of choosing any treatment and the choice of treatment is not deterministic. However, we will illustrate that even though this assumption is violated, the plug-in estimator is still consistent.

Given the treatment and the confounding effects for each decision team, a dichotomous outcome is generated with the following success probability:

$$\begin{array}{r} P(Y=1|V_{1}=v_{1},V_{2}=v_{2},T=t_{i})=0.05\cdot i+0.1\cdot v_{1}+0.1\cdot v_{2}+0.2\;\forall \;i\in \{1,2,3\} \end{array}$$

The coefficients in this process are chosen to somewhat resemble our clinical use-case, presented in Section 5. The ATE is set to 0.05 because this was deemed to be the minimal clinically important effect (non-inferiority margin) on the basis of discussions with clinicians. The coefficients for the confounding effects were set to introduce a similar unobserved confounding effect to what is expected in our clinical use-case. The intercept was chosen to be 0.2 to produce a similar overall average remission rate ( 0.4 ) to what is observed in our clinical data set.

In this simulation, homogeneous treatment effects are assumed for simplicity. Therefore, the conditional expectation in equation (13) should be equal to its marginal counter-part. The ATOs are given as follows:

$$\begin{array}{r} E(Y(t_{i}))=0.05\cdot i+0.1\cdot 0.5+0.1\cdot 0.5+0.2=0.3+0.05\cdot i\;\forall \;i\in \{1,2,3\} \end{array}$$

We simulate data sets from this process with sample sizes $n_{s}$ ranging from 500 to 3000 in steps of 250 . Each sample size step is simulated 5000 times.

### 4.2. Estimation

Given the values $z_{1}-z_{4}$ for the instrument and assuming that all 7 possible adherence sets exist, the following effects are identifiable with the method presented in Section 3:

(16) $$\begin{array}{rl} & E(Y(t_{1})-Y(t_{2})|\{t_{1},t_{3}\}\subseteq A)\\ & E(Y(t_{2})-Y(t_{3})|\{t_{2},t_{3}\}\subseteq A)\\ & E(Y(t_{1})-Y(t_{3})|A=\{t_{1},t_{3}\}) \end{array}$$

One can see that the LATEs for $\left(t_{1},t_{2}\right)$ and $\left(t_{2},t_{3}\right)$ can be identified for the population of decision teams for whom the treatments involved in the comparison are relevant. On the other hand, the LATE for $\left(t_{1},t_{3}\right)$ is identified only for the decision teams for whom these two alternatives but not $t_{2}$ are relevant. Therefore, we expect the latter estimate to have larger bias and variance than the former, even though all estimates are expected to be asymptotically unbiased.

For each simulated experiment, three estimators are calculated for the treatment pairs listed above, denoted as the naive, dichotomous IV (DIV), and CIV estimators. The naive estimator ignores the unobserved confounding effects and only calculates conditional expectations. This corresponds to a real-world situation where classical statistical methods are applied, not addressing unobserved confounding effects with IV analysis. The DIV estimator removes the decision teams choosing treatment alternatives other than the two treatments being compared. It divides the values of the instrument into two groups. One where $t_{i}$ is more encouraged (cheaper) than $t_{j}$ and another where $t_{j}$ is more encouraged than $t_{i}$ . It then performs DIV estimation, as presented in equation (2). This corresponds to a real-world situation where one ignores the existence of relevant alternatives other than the two treatments of interest and compares them using IV analysis. The CIV estimator is our plug-in estimator, presented in Section 3. The mathematical representation of these estimators is given in Supplemental Appendix Subsection B.8.

Since we know that all the estimated effects must lie between −1 and 1 , we remove the simulations that yield IV estimates outside this interval. The naive estimates are necessarily between −1 and 1 per definition. This is, however, not the case for the IV estimates.

### 4.3. Results

For each data set representing a study (with a fixed sample size $n_{s}$ ), we present the median estimate over all repetitions and compare to the true treatment effect difference. We also present the standard deviation of the estimates.

**Figure 3.**
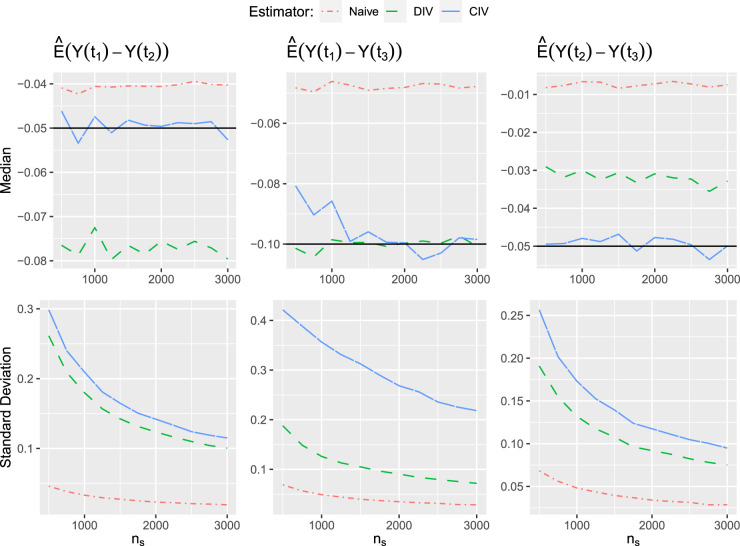
This figure shows the median and the standard deviation over all repetitions from all three estimators in our simulation study. The black line shows the true difference in treatment effects.

The results are shown in Figure 3. For treatment pairs $\left(t_{1},t_{2}\right)$ and $\left(t_{2},t_{3}\right)$ , the CIV estimator clearly outperforms the other two, for which the performance is reduced due to the unobserved confounding effects. In addition, as we previously expected, the variance of the CIV estimator is not substantially larger than that of the DIV estimator. For treatment pair $\left(t_{1},t_{3}\right)$ , the CIV estimator does not seem to perform very well compared to the DIV estimators. Even though the CIV estimator seems to be asymptotically unbiased, it has a much larger variance and larger bias for small population sizes. This lack of performance might be due to that the effect is estimated only for one adherence set.

All in all, this simulation study illustrates in a simple setting that our method can successfully remove the bias caused by the presence of multiple unordered relevant treatment alternatives in IV analysis. However, this advantage disappears if the sub-population for which the LATE is estimated is small, and one could argue that our method is rather inferior to the DIV estimator in that setting, at least if homogeneity can be assumed.

The simple artificial data generating process we have used is unlikely to correspond to any real clinical scenario. Our aim was to provide a simple and transparent process to investigate how the methodology copes with unobserved confounding. Note that the non-parametric theoretical results presented in Section 3 do not pose any restrictions on the data generating process apart from Assumptions (i) to (vi), and the simulation mechanism, while not clinically plausible, does not induce any restrictions on the validity of the results.

**Figure 4.**
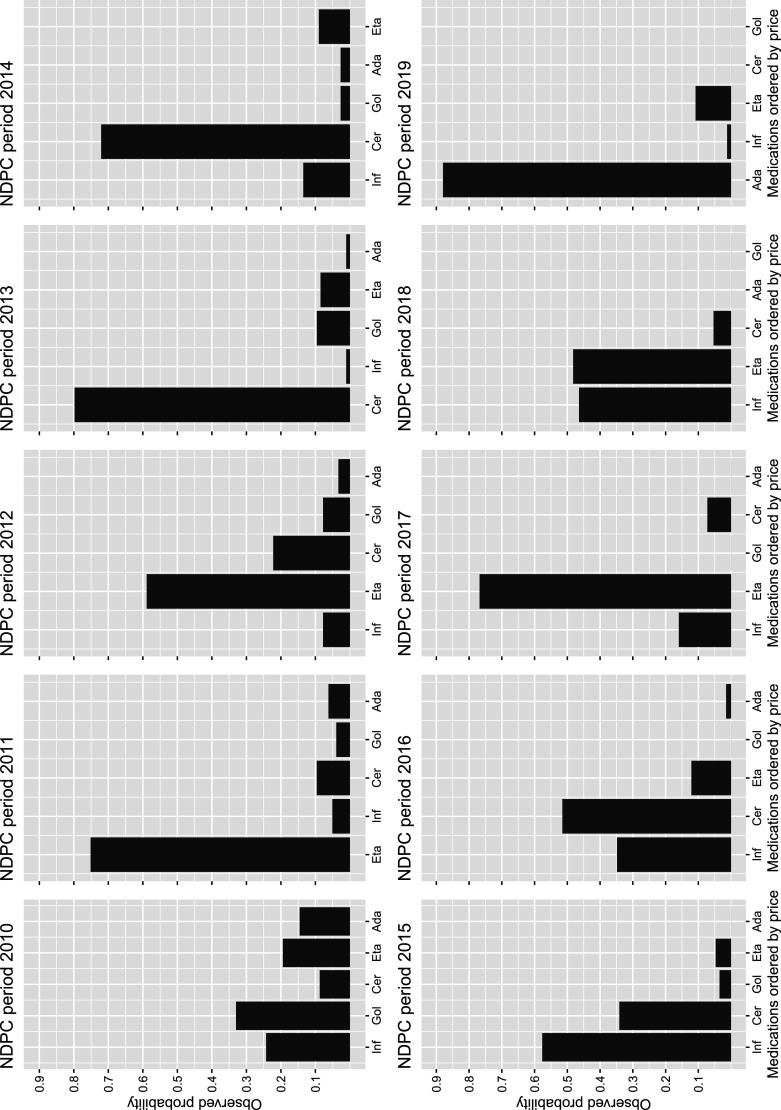
Histograms of portion of patients starting on each of the five TNFis of interest under any given NDPC recommendation. On the *X*-axis, the treatments are sorted according to the NDPC recommendation in the corresponding period. Inf: infliximab; Gol: golimumab; Cer: certolizumab pegol; Eta: etanercept; Ada: adalimumab; TNFis: tumor necrosis factor inhibitors; NDPC: Norwegian Drug Procurement Cooperation.

## 5. Clinical application

As introduced in Subsection 1.3, the method developed in Section 3 is applied for head-to-head comparisons of five TNFis in treatment of RA. The data from 2010 to 2019 is extracted from the NOR-DMARD registry. In this period, the price ordering of the five TNFis of interest changed every year, giving rise to 10 unique values for the instrument. Only patients without a previous history of biologic treatments were included in the analysis as the choice of treatment for these patients is the most affected by the price of the medications. For these patients, we assume that only the five TNFis analyzed in this article are relevant as biologic treatment alternatives. The treatment variable represents only prescription of a drug and non-compliance is ignored. Therefore, the identified LATEs should be interpreted as intention-to-treat effects. The purpose of this section is to illustrate the real-world applicability of our method. Advanced modeling of this particular data set as well as clinical discussions of the results are deemed out of the scope of this work.

In the period 2010–2019, the NDPC arranged a tender in the beginning of every year. The new recommendations were made public in February or March. We refer to the period between two recommendations as a NDPC period, and we label them with the corresponding year in which the recommendation was made.

Clinical remission at three months after treatment start is used as the primary outcome. This is a binary variable derived from the disease activity score based on clinical investigations of 28 joints and the level of C-reactive protein in the blood (DAS28 CRP).^21^ Patients who terminated treatment before three months were considered treatment failures and set as not reaching clinical remission for the assessment at three months. Our method of handling missing data in this data set is described in Supplemental Appendix Subsection B.12.

### 5.1. Descriptive analysis

In total, 934 RA patients were included in the final data set with the distribution over the NDPC periods, as well as baseline characteristics, presented in Table 3. Figure 4 shows the level of adherence to the NDPC recommendations. In this figure, certolizumab and etanercept appear to be more popular choices among decision teams than the other available treatments. This difference in the level of adherence is accounted for in the method presented in Section 3. Otherwise, adherence to the NDPC recommendations appears to be high. The relevance of the NDPC recommendation as an instrument is further discussed in the context of Figure 4 in Supplemental Appendix Subsection B.10.

**Table 3.** Summary statistics for each NDPC period.

Variable ∖ period	2010	2011	2012	2013	2014	2015	2016	2017	2018	2019
Number of patients	103	177	90	94	111	85	66	69	56	83
Mean DAS28 CRP ( 0−9.4 )	4.27	3.97	3.62	3.69	3.82	3.74	3.91	3.69	3.59	3.66
Mean time since diagnosis (years)	6.23	9.16	7.88	8.08	9.99	6.85	6.83	7.68	4.12	4.36
Mean PhGA ( 0−100 )	35.11	32.19	32.14	32.72	34.29	33.67	37.70	33.79	28.47	28.91
Mean age (years)	51.65	53.45	52.68	53.43	53.87	55.53	54.03	53.26	53.60	54.62
Percentage female	77	81	64	72	65	65	70	75	71	69
Percentage anti-CCP positive	76	80	83	73	77	76	76	74	64	64
Percentage current smoker	25	21	11	22	12	23	11	10	20	24

*Note:* All statistics are calculated at baseline omitting any missing observations. PhGA: phycisians global assessment of disease activity; anti-CCP: anti-citrullinated protein antibody; NDPC: Norwegian Drug Procurement Cooperation.

**Figure 5.**
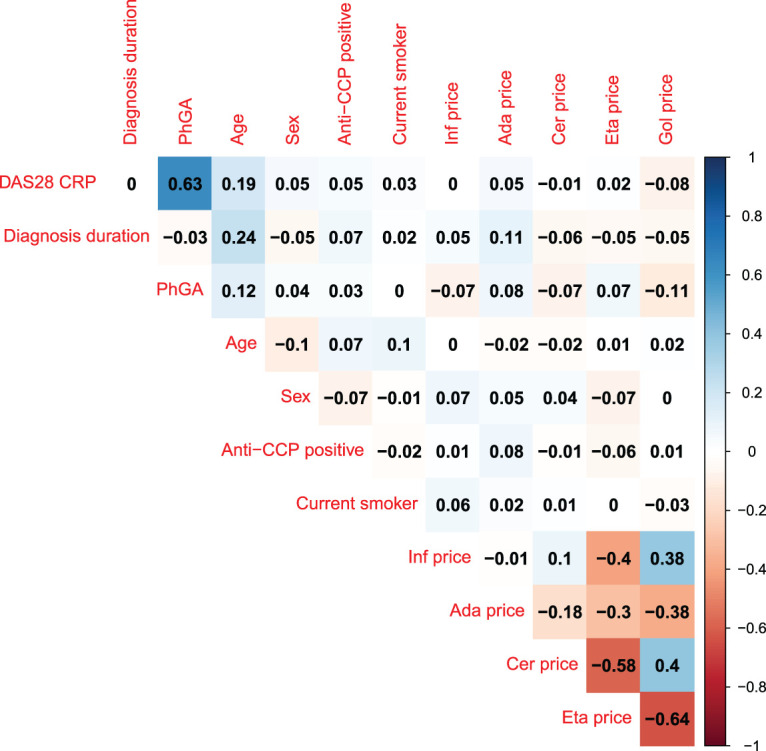
Spearman’s correlation matrix including measured confounding factors and medication prices. Correlations are calculated using pairwise complete observations. PhGA: phycisians global assessment of disease activity; anti-CCP: anti-citrullinated protein antibody; Inf: infliximab; Gol: golimumab; Cer: certolizumab pegol; Eta: etanercept; Ada: adalimumab.

In Figure 5, one can see the Spearman’s correlation between measured known confounding factors and the instrument. The correlation coefficients between measured confounding factors and the instrument (medication prices) do not exceed 0.11 . This low level of correlation further supports our assumption that the NDPC recommendation does not affect the outcome except through its effect on the treatment. While the exogeneity of the NDPC recommendation is a reasonable assumption, we have only observed 10 out of 5!=120 possible realizations of the instrument. So, while the estimator is consistent under the given assumptions, it might be inefficient. One way of increasing the efficiency is to introduce more information, for example, through covariate information. This should be done using the methodology introduced in Subsection 3.6 to avoid opening a causal pathway. As a robustness analysis, we applied the method developed in Supplemental Appendix Subsection B.6 to adjust for DAS28 CRP at baseline. We assume that this variable summarizes the state of the patient at inclusion. Further model specifications and results of this sensitivity analysis are provided in Supplemental Appendix Subsection B.13.

Not all the data available in NOR-DMARD was used in this analysis. Data prior to 2010 was not used due to absence of some of the TNFis of interest from the NDPC tender. Data after 2019 was not used due to possible interference with the COVID-19 pandemic, since it could be argued that decision teams starting treatment in the pandemic were not comparable to the decision teams starting treatment prior to the pandemic.

### 5.2. Results

The method presented in Section 3 was applied to the NOR-DMARD data set. Bootstrapping with 2500 replications was performed. Bootstrapping estimates were capped at ±1 , meaning that replications that produced effects outside this interval were removed. To increase stability of the point-estimates, we report the bootstrap median estimates and 95% confidence intervals (CIs). In Table 4, we present the sub-populations for which treatment effects are identifiable, together with their estimated probability. Note that equation (12) is used to estimate this probability, which poses no restriction on the sign of the estimates. Therefore, probabilities that are close to zero might be estimated to be negative due to random variations in the data. In addition, this probability can be estimated using either of the treatment alternatives involved in the comparison in the right-hand side of equation (12). Both estimates are presented in Table 4. In this table, we also present the estimates of all identifiable LATEs, together with their 95% CIs.

**Table 4.** The first column lists all identifiable LATEs specifying their corresponding sub-population.

LATE	$\hat{P}(\Sigma )$	$\hat{E}(.)$	CI	n
E(Y(Inf)−Y(Cer)|{Inf,Cer}⊆A)	0.28,0.27	0.55	(0.14,0.95)	2356
E(Y(Inf)−Y(Eta)|{Inf,Eta}⊆A)	0.24,0.14	0.10	(-0.49,0.77)	2451
E(Y(Ada)−Y(Eta)|A={Ada,Eta})	-0.01,0.02	0.08	(-0.89,0.91)	1621
E(Y(Ada)−Y(Gol)|A={Ada,Gol})	-0.04,0.04	-0.57	(-0.84,-0.23)	2500
E(Y(Cer)−Y(Eta)|{Cer,Eta}⊆A⊆{Ada,Cer,Eta,Gol})	0.40,0.49	0.21	(0.04,0.38)	2500
E(Y(Cer)−Y(Gol)|{Cer,Gol}⊆A⊆{Ada,Cer,Gol})	0.05,-0.04	-0.73	(-0.99,0.36)	962
E(Y(Cer)−Y(Gol)|{Cer,Gol}⊆A⊆{Ada,Cer,Eta,Gol})	0.44,0.28	0.30	(0.09,0.52)	2500
E(Y(Cer)−Y(Gol)|{Cer,Gol}⊆A⊆{Cer,Eta,Gol})	0.39,0.32	0.37	(0.16,0.61)	2500
E(Y(Eta)−Y(Gol)|{Eta,Gol}⊆A⊆{Ada,Eta,Gol})	0.01,0.01	0.07	(-0.91,0.93)	556

*Note*: Both estimates for the probability of the sub-populations are presented in $\hat{P}(\Sigma )$ . The estimate before and after comma are calculated using the first and the second treatment alternative in the right-hand side of equation (12), respectively. The LATE estimates are given under $\hat{E}(.)$ . CIs are the 95% bootstrapping confidence interval for these estimates. The number of replications that yielded an estimate in the ±1 interval for each LATE are reported as n . Inf: infliximab; Gol: golimumab; Cer: certolizumab pegol; Eta: etanercept; Ada: adalimumab; LATE: local average treatment effect.

Even though most CIs are very wide, some conclusions can be inferred from the results presented in Table 4. For instance, the evidence shows that golimumab is better than adalimumab for decision teams that consider **only** these two treatment alternatives as relevant. There is some indication that infliximab might be better than certolizumab for decision teams that consider both treatment alternatives as relevant. For decision teams that consider infliximab as irrelevant and both certolizumab and etanercept as relevant, the latter two treatment alternatives likely have similar effects. Additionally, for decision teams that consider infliximab as irrelevant and both certolizumab and golimumab as relevant, certolizumab is better than golimumab. These sub-populations could be described with the help of observed covariates and further discussed from a clinical point of view. This is, however, out of the scope of this work.

## 6. Discussion and conclusion

In this article, we have presented an IV framework to address the methodological challenges that arise in estimating LATEs with the help of an instrument when multiple relevant treatment alternatives are present. Note that our methodology does not address general concerns regarding IV analyses, as these are explored extensively for the case of dichotomous treatment variable. The same challenges and solutions apply in our case. We assume that the instrument is at least conditionally exogenous. A violation of this assumption will break consistency. Therefore, IV analysis shouldn’t be seen as an alternative to causal inference. One should still think carefully about all potential causal pathways and block all backdoor paths between the instrument and the outcome.

The efficiency of our IV estimator is strongly influenced by the strength of the instrument. In the dichotomous case, the strength of the instrumental effect can be measured by estimating the probability of compliance. This is the denominator in the dichotomous IV estimator. The strength of the instrumental effect in a given comparison in our case can also be measured by the magnitude of the denominator in our proposed estimator. If the denominator is small (e.g. below 0.1), the generalizability of the result should be doubted. Additionally, the estimator will likely be too inefficient for the results to be informative given a finite sample size. However, there exist solutions for increasing efficiency in the case of weak instruments, some of which could be generalized to be used within our framework.^22^

Our main contribution has been to identify and present a set of assumptions which enable effect identification while still being plausible in many real-world situations. The main difference between our method and the methods developed by Heckman and Pinto^15^ is in the assumptions. While Heckman and Pinto^15^ systematically dismissed principal strata by assuming unordered monotonicity, we achieve this goal by introducing adherence sets and requiring the instrument to be ordinal. In addition, Heckman and Pinto^15^ provided an analytic solution to equation (10) given unordered monotonicity. In our case, this has not been achieved. Instead we rely on solving this equation numerically. By introducing the adherence set as a balancing score, we were able to reduce the number of principal strata systematically based on choice theoretical axioms. In addition, we illustrated that by solving $b_{t}K_{t}=0$ numerically, one might be able to achieve identification for some sub-populations without needing to assume Heckman and Pinto’s^15^ unordered monotonicity.

A strength of this article is that it is motivated by and applied to a real-world data set to inform a highly interesting clinical causal question. Detailed clinical discussions of the results is, however, out of the scope of this article. We illustrate that the method is applicable in this and other similar real-world situations. Further investigations are needed for the clinical results to be useful. Firstly, (conditional) exogeneity remains to be sufficiently justified in a clinical discussion. This assumption can be quantitatively tested in equation (29). Secondly, further clinical discussions might rule out the existence of some adherence sets, which could increase the efficiency of the estimator. The sub-populations for which LATEs are estimated should be clinically interpreted and the generalizablity of the results should be assessed accordingly. A quantitative description of these sub-populations can be provided by equation (15) to assist clinicians in describing the patient populations for whom the results are valid. Further discussion points, including which target trials^23^ can be emulated by this framework, is given in Section B of the Supplemental Material.

In conclusion, we have provided an additional tool in the toolbox for causal inference when comparing more than two interventions influenced by an exogenous factor. While this is a potential powerful tool, care should be taken in the application. The assumptions presented in this article might not hold in all similar applications. We recommend thinking choice theoretically when considering similar situations, as illustrated previously by Cheng and Small,^11^ Hull,^13^ Blackwell,^14^ and Heckman and Pinto.^15^

## Supplemental Material

sj-pdf-1-smm-10.1177_09622802241281960 - Supplemental material for Instrumental variable analysis with categorical treatment Supplemental material, sj-pdf-1-smm-10.1177_09622802241281960 for Instrumental variable analysis with categorical treatment by Amir Aamodt Kazemi and Inge Christoffer Olsen in Statistical Methods in Medical Research
