# Supplementary material for: Instrumental variable analysis with categorical treatment
Source: Stat Methods Med Res. 2024 Oct 30;33(11-12):2043–61. doi: 10.1177/09622802241281960 (PMC11577691; doi:10.1177/09622802241281960)
Supplement: sj-pdf-1-smm-10.1177_09622802241281960 - Supplemental material for Instrumental variable analysis with categorical treatment [file sj-pdf-1-smm-10.1177_09622802241281960.pdf]

SUPPLEMENTARY MATERIAL

A Proofs

A.1 Proof of Proposition 1

Every decision team must necessarily belong to one and only one of the four groups listed in Table 1. Further, the principal stratum, S, and ϵ are independent given Assumptions (i) - (iii). Then we have:

$$
\begin{aligned}
E(Y|Z=z_a) =& P(AT_a)E(Y_{AT_a}(a))+ \\
& P(NT_a)E(Y_{AT_b}(b))+ \\
& P(C)E(Y_C(a))+ \\
& P(D)E(Y_D(b)) \\
E(Y|Z=z_b) =& P(AT_a)E(Y_{AT_a}(a))+ \\
& P(AT_b)E(Y_{AT_b}(b))+ \\
& P(C)E(Y_C(b))+ \\
& P(D)E(Y_D(a)) \\
E(Y|Z=z_a)-&E(Y|Z=z_b) = \\
& P(C)E(Y_C(a)-Y_C(b))- \\
& P(D)E(Y_D(a)-Y_D(b)), \qquad (17)
\end{aligned}
$$

where for simplicity, we have denoted $E(Y(t)|S=s)$ as $E(Y_s(t))$ and $P(S=s)$ as $P(s)$ for $s \in \{AT_a, AT_b, C, D\}$. Looking at the probabilities we have:

$$
\begin{aligned}
&P(T=a|Z=z_a) = P(AT) + P(C) \ \& \\
&P(T=a|Z=z_b) = P(AT) + P(D) \\
&\Rightarrow P(T=a|Z=z_a) - P(T=a|Z=z_b) = P(C) - P(D). \qquad (18)
\end{aligned}
$$

If homogeneity is assumed, then:

$$
E(Y_C(a)-Y_C(b)) = E(Y_D(a)-Y_D(b)) = E(Y(a)-Y(b)). \qquad (19)
$$

Equation 2 can then be derived from Equations 17 - 19.

If monotonicity is assumed then:

$$
P(D) = 0. \qquad (20)
$$

Equation 3 can then be derived from Equations 17, 18 and 20.

A.2 Proof of Remark 1

In the context of Table 1, if we try to apply the same mathematical derivation as for the dichotomous case in Subsection A.1, we obtain the following for the numerator of the estimator provided in Equation 2:

$$
\begin{aligned}
E(Y|Z=z_b) - E(Y|Z=z_a) =& \\
& P(NT_a^c)E(Y(b)-Y(c))+ \\
& P(NT_b^c)E(Y(c)-Y(a))+ \\
& (P(NT_c^a)+P(NT_c^b)+P(C))E(Y(b)-Y(a))
\end{aligned}
$$

One can see in the equation above that $Y(c)$ appears in the expression of the numerator in the estimator for the effect $E(Y(b)-Y(a))$. Therefore, in the presence of a third treatment alternative, this simple generalization of the dichotomous IV estimator doesn't estimate $E(Y(b)-Y(a))$.

A.3 Proof of Lemma 1

From IC 1 follows:

$$
E(Y(t)|A) = E(Y(t)|A, T=t)
$$

The right-hand side of this equation can be rewritten as:

$$
\begin{aligned}
E(Y(t)|A, T=t) &= \sum_{t'} E(Y(t') \cdot 1[T=t']|A, T=t) \\
&= E(\sum_{t'} Y(t') \cdot 1[T=t']|A, T=t) \\
&= E(Y|A, T=t),
\end{aligned}
$$

where the last equation follows from consistency (Equation 1).

A.4 Proof of Lemma 2

The LATO can be written as:

$$
E(Y(t)|w \in \Sigma) = \frac{E(Y(t) \cdot 1[w \in \Sigma])}{P(w \in \Sigma)}. \qquad (21)
$$

Note that:

$$
w \in \Sigma \Leftrightarrow a \in \Pi \text{ and } \Omega_a \cap \Omega_{a'} = \emptyset.
$$

Therefore, Equation 21 can be rewritten as:

$$
\begin{aligned}
E(Y(t)|w \in \Sigma) &= \frac{E(Y(t) \cdot 1[a \in \Pi])}{P(a \in \Pi)} \\
&= \frac{\sum_{a \in \Pi} E(Y(t) \cdot 1[A=a])}{P(a \in \Pi)} \\
&= \frac{\sum_{a \in \Pi} E(Y(t)|A=a)P(A=a)}{P(a \in \Pi)}.
\end{aligned}
$$

Applying Lemma 1 to this equation, we have:

$$
\begin{aligned}
&E(Y(t)|w \in \Sigma) = \\
&\quad \frac{1}{P(a \in \Pi)} \sum_{a \in \Pi} E(Y|A=a, T=t)P(A=a).
\end{aligned}
$$

Note that Lemma 1 is applicable here because Π is defined such that positivity is ensured, namely:

$$
\forall a \in \Pi, \ t \in a \Rightarrow P(T=t|A=a) > 0.
$$

A.5 Proof of Proposition 2

These equations can be proven following the same steps as in the proof given in Supplemental Material Appendix A.2 in Heckman and Pinto [15]. Note that from Assumption (vi), we have:

$$
P(T=t|A=a, Z=z) = 1[T=t|A=a, Z=z]. \qquad (22)
$$

From Equation 22 and IC 2, we have:

$$
\begin{aligned}
&P(T=t|Z=z) = \\
&\quad \sum_a P(T=t|A=a, Z=z)P(A=a|Z=z) = \\
&\quad \sum_a P(T=t|A=a, Z=z)P(A=a) = \\
&\quad \sum_a 1[T=t|A=a, Z=z]P(A=a)
\end{aligned}
$$

To prove Equation 7, we need to first prove a lemma.

Lemma 4. *For any three variables, Y, T and Z, we have:*

$$E(Y|Z=z,T=t)P(T=t|Z=z) = E(Y\cdot 1[T=t]|Z=z).$$

Proof.

$$\begin{aligned}&E(Y|Z=z,T=t)P(T=t|Z=z)\\ &\quad= 0\cdot E(Y|Z=z,T\neq t)P(T\neq t|Z=z)\\ &\quad+E(Y|Z=z,T=t)P(T=t|Z=z)\\ &\quad= E(Y\cdot 1[T=t]|Z=z,T\neq t)P(T\neq t|Z=z)\\ &\quad+E(Y\cdot 1[T=t]|Z=z,T=t)P(T=t|Z=z)\\ &\quad= E(Y\cdot 1[T=t]|Z=z)\end{aligned}$$

The last step comes from the law of total expectation.

Also from Equation 22, IC 2 and IC 3:

$$\begin{aligned}&E(Y|Z=z,T=t)P(T=t|Z=z)=E(Y\cdot 1[T=t]|Z=z)\\ &=\sum_a E(Y\cdot 1[T=t]|Z=z,A=a)P(A=a|Z=z)\\ &=\sum_a E(Y\cdot 1[T=t]|Z=z,A=a)P(A=a)\\ &=\sum_a P(A=a)E(Y|Z=z,T=t,A=a)\\ &\qquad P(T=t|Z=z,A=a)\\ &=\sum_a 1[T=t|Z=z,A=a]\\ &\qquad E(Y|Z=z,T=t,A=a)P(A=a)\\ &=\sum_a 1[T=t|Z=z,A=a]E(Y|T=t,A=a)P(A=a),\end{aligned}$$

where in the first and the fourth equations, we have applied Lemma 4. The third and last equation follow from IC 2 and 3, respectively. The fifth equation follows from Equation 22, while the second equation is the law of total expectation.

A.6 Proof of Theorem 1

Keeping in mind the definition of K_t and b_t in Definition 13, pseudo-inverting Equations 8 and 9 will give:

$$Q_A(t) = B_t^+ Q_Z(t) + K_t\lambda \tag{23}$$
$$P_A = B_t^+ P_Z(t) + K_t\mu, \tag{24}$$

where λ and μ are arbitrary vectors. Multiplying both sides of Equations 23 and 24 by b_t gives:

$$b_tQ_A(t) = b_tB_t^+Q_Z(t)$$
$$b_tP_A = b_tB_t^+P_Z(t),$$

which is the first part of the theorem. In these equations, we have identified $b_tQ_A(t)$ and b_tP_A for all binary vectors b_t that satisfy Equation 10.

On the other hand, applying Lemma 2 to $\Pi(b_t)$ and $\Sigma(b_t)$ as defined in Definition 14, we have:

$$E(Y(t)|w\in\Sigma(b_t)) = \frac{1}{P(a\in\Pi(b_t))}\sum_{a\in\Pi(b_t)}E(Y|A=a,T=t)P(A=a). \tag{25}$$

One can see that the quantities on the right-hand side of Equation 25 are precisely the quantities identified in Equations 11 and 12. Namely:

$$b_tQ_A(t) = \sum_{a\in\Pi(b_t)}E(Y|A=a,T=t)P(A=a)$$
$$b_tP_A = \sum_{a\in\Pi(b_t)}P(A=a) = P(a\in\Pi(b_t)).$$

In conclusion, the LATO of a treatment t is identified for all sub-populations $\Sigma(b_t)$ corresponding to the solutions of Equation 10, according to Definition 14. These effects can be written in terms of observables as:

$$E(Y(t)|w\in\Sigma(b_t)) = \frac{b_tB_t^+Q_Z(t)}{b_tB_t^+P_Z(t)}.$$

For head-to-head comparison of two treatment alternatives, we can substract the LATO of the one from that of the other for the same sub-population to arrive at the identity stated in the theorem.

Note, there is still one loose end. For Lemma 2 to be applicable here, we need to ensure positivity. Positivity in this equation means:

$$P(T=t|A=a)>0,$$

for all t and a such that $a\in\Pi(b_t)$. Applying the definition of adherence set (Definition 10), we need to prove:

$$\forall a \mid a\in\Pi(b_t), t\in a.$$

This can be inferred from our assumptions. We prove this in the following lemma:

Lemma 5. *For any treatment t, if $a\in\Pi(b_t)$ for any solution, b_t, to Equation 10, then $t\in a$.*

Proof. Without loss of generality, assume a is the first adherence set corresponding to the first column of B_t and the first element of b_t. We will apply proof by contradiction. Assume $t\notin a$. Following Definition 10, this means under no values of the instrument, t would be chosen by the decision teams in Ω_a. Therefore, the first column of B_t is necessarily all zeros:

$$B_t[:,1]=0 \Rightarrow (B_t^+B_t)[:,1]=0 \Rightarrow K_t[:,1]=[1,0,0,...,0]$$

On the other hand, since $a\in\Pi(b_t)$, we know that $b_t[1]=1$. Therefore:

$$(b_tK_t)[1]=1,$$

but we know that $b_tK_t=0$ per definition. This is a contradiction. We can therefore conclude that $t\in a$.

We have now shown positivity and the proof is completed.

A.7 Proof of Corollary 1

Denote variables Υ and Φ and function $f(\upsilon,\phi)$ as:

$$\Upsilon = bB_t^+Q_Z(t),\qquad \Phi = bB_t^+P_Z(t),\qquad f(\upsilon,\phi)=\frac{\upsilon}{\phi}.$$

Then we want to show:

$$E(f(\hat\Upsilon,\hat\Phi) = f(\Upsilon,\Phi)+O(n^{-1}),$$

where n is the number of observations. First note that:

$$E(\hat{\Upsilon}) = E(bB_t^+\hat{Q}_Z(t)) = bB_t^+E(\hat{Q}_Z(t)) = bB_t^+Q_Z(t)$$
$$E(\hat{\Phi}) = E(bB_t^+\hat{P}_Z(t)) = bB_t^+E(\hat{P}_Z(t)) = bB_t^+P_Z(t).$$

In other words, $\hat{\Upsilon}$ and $\hat{\Phi}$ are unbiased. Applying the second-order Taylor expansion around Υ and Φ, we have:

$$\begin{aligned} E(f(\hat{\Upsilon},\hat{\Phi})) &= f(\Upsilon,\Phi) + f'_\upsilon(\Upsilon,\Phi)E((\hat{\Upsilon}-\Upsilon)) \\ &+ f'_\phi(\Upsilon,\Phi)E((\hat{\Phi}-\Phi)) \\ &+ \frac{1}{2}(f''_{\upsilon\upsilon}(\Upsilon,\Phi)E((\hat{\Upsilon}-\Upsilon)^2) \\ &+ f''_{\phi\phi}(\Upsilon,\Phi)E((\hat{\Phi}-\Phi)^2)) \\ &+ f''_{\upsilon\phi}(\Upsilon,\Phi)E((\hat{\Upsilon}-\Upsilon)(\hat{\Phi}-\Phi)) + R. \end{aligned}$$

Since $\hat{\Upsilon}$ and $\hat{\Phi}$ are unbiased, the first order terms vanish. Also $f''_{\upsilon\upsilon} = 0$. The other two second order terms, however, remain. Therefore:

$$E(f(\hat{\Upsilon},\hat{\Phi})) = f(\Upsilon,\Phi) + O(Var(\hat{\Phi})) + O(Cov(\hat{\Upsilon},\hat{\Phi})).$$

From the law of large numbers and the assumption that the number of observations is homogeneous across the values of Z, we have:

$$O(Var(\hat{\Phi})) = O(Cov(\hat{\Upsilon},\hat{\Phi})) = O(n^{-1}).$$

Therefore:

$$E(f(\hat{\Upsilon},\hat{\Phi})) = f(\Upsilon,\Phi) + O(n^{-1})$$

and the proof is completed.

B Further Discussions and Examples

B.1 Clinical Implications of the Assumptions

In our clinical use-case, Assumptions (i)-(iii) mean:

(i) The choice of treatment by decision team w only depends on the price-ordered treatment list in the NDPC period they start the treatment, the confounding factors such as patient's comorbidities, physicians preferences, etc and a random residual. The confounding factors might be observed or unobserved.

(ii) The outcome for decision team w after three months only depends on the treatment choice by the decision team, the confounding factors and a random residual. This means the method allows for two decision teams that start treatment in the same year and have exactly the same values for all observed and unobserved confounding effects to achieve different outcomes, only at random.

(iii) The prices of the treatments, the confounding effects and the random residuals are mutually independent from each other.

B.2 Adherence Sets in a Clinical Context

Here, we give a few examples of what adherence sets are realistic in our clinical use-case. Let's consider a decision team w where the patient has uveitis. Therefore, the physician considers etanercept as not being a relevant treatment alternative for the patient. We would then have:

$$A = \{\text{infliximab, adalimumab, certolizumab, golimumab}\}.$$

If the physician has a strong preference for the three original TNF-inhibitors, then:

$$A = \{\text{adalimumab, etanercept, infliximab}\}.$$

In our method, we basically apply the weak axiom of revealed preference (WARP) from choice theory to our observed data to make inferences about the adherence set, which is an unobserved variable. WARP states that decision makers are consistent with their preferences. For instance, if a decision team chooses the second cheapest medication when etanercept is the cheapest alternative, they have revealed that they have a preference for not choosing etanercept. WARP states that if they didn't choose etanercept when it was the cheapest option, they would never choose etanercept. We can then infer that etanercept was not in the adherence set of that decision team. We can also infer that the chosen medication indeed was in that set.

B.3 Further Discussion of the Choice Model

Essentially, Assumptions (i)-(vi), together with the choice function defined in Equation 4, mean that, in our clinical use-case, each decision team first decide on a subset of the possible treatments that are relevant for them, and then look at the price list and select the cheapest alternative in that subset. The important point here is that the set of relevant alternatives must be independent from the price list.

Example 1. Consider the dichotomous case and apply Assumptions (i)-(vi). The adherence set can only take three different values, namely $a_1 = \{a\}$, $a_2 = \{b\}$ and $a_3 = \{a, b\}$. The instrument can only take the two values $z_a = (a, b)$ and $z_b = (b, a)$, which means that for each decision team, either the treatment is encouraged or discouraged. There exist three different groups of decision teams, namely:

1 $\Omega_1 = \{w|A = a_1\}$: Always-takers of a. This group will take treatment a regardless of the value of the instrument.

2 $\Omega_2 = \{w|A = a_2\}$: Never-takers of a. This group will not take treatment a regardless of the value of the instrument.

3 $\Omega_3 = \{w|A = a_3\}$: Compliers. This group will take treatment a when $Z = z_a$, and not take treatment a when $Z = z_b$.

In other words, we assume there exists no group of decision teams that choose treatment a when $Z = z_b$ and not take treatment a when $Z = z_a$, which means no defiers. The trick is the way g is defined in Equation 4. Always selecting the most encouraged treatment, rules out the existence of defiers in the dichotomous case, which is the same as monotonicity.

B.4 Examples

Here we will land from mathematical abstractions onto some hypothetical examples to help the readers regain their

intuition. We apply our method to the simplest case, which is a setting with a dichotomous treatment. Then, $\Sigma(b)$ is exactly the set of all compliers and Equation 14 is equivalent to the dichotomous IV identity. Let's go through it together. Consider the dichotomous example presented in Example 1. Then we have:

$$B_a = \begin{bmatrix} 1 & 0 & 1 \\ 1 & 0 & 0 \end{bmatrix}, B_b = \begin{bmatrix} 0 & 1 & 0 \\ 0 & 1 & 1 \end{bmatrix}$$

$$B_a^+ = \begin{bmatrix} 0 & 1 \\ 0 & 0 \\ 1 & -1 \end{bmatrix}, B_b^+ = \begin{bmatrix} 0 & 0 \\ 1 & 0 \\ -1 & 1 \end{bmatrix}$$

$$K_a = \begin{bmatrix} 0 & 0 & 0 \\ 0 & 1 & 0 \\ 0 & 0 & 0 \end{bmatrix}, K_b = \begin{bmatrix} 1 & 0 & 0 \\ 0 & 0 & 0 \\ 0 & 0 & 0 \end{bmatrix}.$$

We see that B_t^+ is a pseudo-inverse to B_t because it fulfills the equation $B_t B_t^+ B_t = B_t$. The only binary non-zero common solution to Equation 10 is $b = [0, 0, 1]$. This corresponds to $\Pi(b) = \{a_3\}$ and $\Sigma(b) = \Omega_3$, which as previously discussed in Example 1, is the set of compliers. The LATE estimate provided in Equation 14 is then:

$$\begin{aligned} &E(Y(a) - Y(b)|w \in \Omega_3) \\ &= \frac{[1,-1] \times Q_Z(a)}{[1,-1] \times P_Z(a)} - \frac{[-1,1] \times Q_Z(b)}{[-1,1] \times P_Z(b)} \\ &= \frac{E(Y \cdot 1[T=a]|Z=z_a) - E(Y \cdot 1[T=a]|Z=z_b)}{P(T=a|Z=z_a) - P(T=a|Z=z_b)} \\ &- \frac{E(Y \cdot 1[T=b]|Z=z_b) - E(Y \cdot 1[T=b]|Z=z_a)}{P(T=b|Z=z_b) - P(T=b|Z=z_a)}. \end{aligned}$$

Taking advantage of

$$P(T = a|Z) = 1 - P(T = b|Z),$$

one can show:

$$E(Y(a) - Y(b)|w \in \Omega_3) = \frac{E(Y|Z=z_a) - E(Y|Z=z_b)}{P(T=a|Z=z_a) - P(T=a|Z=z_b)},$$

which is the dichotomous IV identity!

We promised that our framework provides a natural gateway for clinical assumptions to enter and contribute to the analysis. We will illustrate this in an example with three possible treatment alternatives, a, b and c. Without any further clinical assumptions, there exist $2^3 - 1 = 7$ adherence sets. Even though some LATEs should be identifiable with data on enough values of the instrument without the need of additional clinical assumptions, for the sake of illustration, let's say that our clinician colleagues can tell us that there is a consensus in the field about treatment a and c being very similar. Therefore, if a decision team adheres to a recommendation encouraging them to take treatment c, there is no reason to believe that they might refuse to take treatment a if a was encouraged and vice versa. Based on this assumption, we can remove $\{a\}$, $\{c\}$, $\{a, b\}$ and $\{b, c\}$ from the set of possible adherence sets, leaving us with only 3 of them. Let's numerate them as following:

- $a_1 = \{b\}$
- $a_2 = \{a, c\}$
- $a_3 = \{a, b, c\}$

The mathematical expression of this clinical assumption is:

$$\begin{aligned} P(A = \{a\}) = P(A = \{c\}) = \\ P(A = \{a, b\}) = P(A = \{b, c\}) = 0. \end{aligned}$$

Let's say that we have data only under two values of the instrument, namely $z_1 = (a, b, c)$ and $z_2 = (c, b, a)$. With this limited amount of data, no LATEs would have been identifiable without the clinical assumption. In other words, assuming that all 7 possible adherence sets exist, there is no vector b that satisfies $bK_t = bK_{t'} = 0$ for any pair of $t \neq t'$. However, applying the additional clinical assumption, we are able to identify $E(Y(c) - Y(a)|w \in \Omega_2 \cup \Omega_3)$. This allows us to test whether or not treatments a and c actually yield similar response rates in the portion of the population that adheres to both. We will derive the estimator for the local average difference in the treatment effect in this case.

$$B_a = \begin{bmatrix} 0 & 1 & 1 \\ 0 & 0 & 0 \end{bmatrix}, B_c = \begin{bmatrix} 0 & 0 & 0 \\ 0 & 1 & 1 \end{bmatrix}$$

$$B_a^+ = \begin{bmatrix} 0 & 0 \\ 0.5 & 0 \\ 0.5 & 0 \end{bmatrix}, B_c^+ = \begin{bmatrix} 0 & 0 \\ 0 & 0.5 \\ 0 & 0.5 \end{bmatrix}$$

$$K_a = \begin{bmatrix} 1 & 0 & 0 \\ 0 & 0.5 & -0.5 \\ 0 & -0.5 & 0.5 \end{bmatrix}, K_c = \begin{bmatrix} 1 & 0 & 0 \\ 0 & 0.5 & -0.5 \\ 0 & -0.5 & 0.5 \end{bmatrix}.$$

One can see that the only non-zero binary common solution to Equation 10 is $b = [0, 1, 1]$. This corresponds to $\Pi(b) = \{a_2, a_3\}$ and $\Sigma(b) = \Omega_2 \cup \Omega_3$, which is the set of decision teams that adhere to both a and c. The LATE estimate provided in Equation 14 is then:

$$\begin{aligned} &E(Y(c) - Y(a)|w \in \Omega_2 \cup \Omega_3) = \\ &\frac{[0,1] \times Q_Z(c)}{[0,1] \times P_Z(c)} - \frac{[1,0] \times Q_Z(a)}{[1,0] \times P_Z(a)} = \\ &\frac{E(Y \cdot 1[T=c]|Z=z_2)}{P(T=c|Z=z_2)} - \frac{E(Y \cdot 1[T=a)|Z=z_1]}{P(T=a|Z=z_1)}. \end{aligned}$$

Taking advantage of

$$E(Y \cdot 1[T = t]|Z) = E(Y|T = t, Z) \times P(T = t|Z),$$

one can show:

$$\begin{aligned} &E(Y(c) - Y(a)|w \in \Omega_2 \cup \Omega_3) = \\ &E(Y|Z = z_2, T = c) - E(Y|Z = z_1, T = a). \end{aligned}$$

B.5 Comparison to Unordered Monotonicity

In order to distinguish our set of assumptions from unordered monotonicity, and to show that our assumptions are more applicable, we will present a plausible example obeying Assumptions (i)-(vi) and choice function defined in Equation 4 which violates unordered monotonicity.

Example 2. Consider an experiment with three treatment alternatives, namely a, b and c. Assume that Z takes the two following values:

- $z_1 = (a, b, c)$
- $z_2 = (c, b, a)$

Now consider the two following groups of decision teams:

- $\Omega_{\{a,b\}} = \{w|A = \{a, b\}\}$
- $\Omega_{\{b,c\}} = \{w|A = \{b, c\}\}$

Decision teams in $\Omega_{\{a,b\}}$ would choose treatment a under z_1 and treatment b under z_2, while decision teams in $\Omega_{\{b,c\}}$ would choose treatment b under z_1 and treatment c under z_2. This means decision teams in $\Omega_{\{a,b\}}$ move towards treatment b if moved from z_1 to z_2, while decision teams in $\Omega_{\{b,c\}}$ move away from treatment b if moved from z_1 to z_2. This is a two-way flow of decision teams in and out of treatment b, which violates unordered monotonicity. This scenario is however allowed under our set of assumptions.

The case presented in Example 2 can be analyzed by our method because unlike unordered monotonicity, our set of assumptions (Assumptions (iv)-(vi)) don't limit the flow of probabilities between different values of instruments to only one direction.

To further differentiate our set of assumptions from unordered monotonicity, we will now present an example that violates unordered monotonicity, where our assumptions give rise to identifiable LATEs. Building on the previous example, we have $z_1 = (a, b, c)$ and $z_2 = (c, b, a)$. As we discussed in Example 2, in the existence of all of the adherence sets, these values for the instrument violate unordered monotonicity. We noted in Subsection B.4 that without further restrictions on the adherence sets, no effects are identifiable given only these two values for the instrument. Now assume data is available under two more values of the instrument, namely $z_3 = (c, a, b)$ and $z_4 = (b, a, c)$. Of course, there are now subsets of $\{z_1, z_2, z_3, z_4\}$ where unordered monotonicity holds even assuming that all adherence sets exist, e.g. $\{z_1, z_4\}$. Taking advantage of these subsets, some LATEs are identifiable both by our and Heckman and Pinto [15]'s methodology, e.g. $E(Y(b) - Y(a)|\{a, b\} \subseteq A)$. However, the full set of z_is still violates unordered monotonicity. In that case, in the existence of all adherence sets and taking advantage of all available values for the instrument, one can show that our assumptions give rise to additional identifiable LATEs, e.g. $E(Y(b) - Y(a)|A = \{a, b, c\})$.

B.6 Generalization of the Theory to Incorporate Conditional Instruments

In this subsection we will aim at providing a tool for breaking backdoor causal pathways between the instrument and the outcome by replacing the independence assumptions with its conditional version and adding a positivity assumption.

Assumption With C denoting confounding factors affecting the instrument and the outcome, replace Assumption (iii) with:

(iii)′ V, Z, ϵ and δ are mutually independent conditioned on C.

Add the following positivity assumption:

(vii) $P(A = a|C = c) > 0 \ \forall \ a, c$: All adherence sets are possible under all values of C.

Note that the effect of C on T must go through either A or Z. Therefore, we have the following independence conditions:

4. $Y(t) \perp\!\!\!\perp T|(A, C)$
5. $A \perp\!\!\!\perp Z|C$
6. $Y \perp\!\!\!\perp Z|(A, T, C)$
7. $T \perp\!\!\!\perp C|(A, Z)$

Note that the conditional version of the assumptions and independence conditions listed here are valid, among others, in the context of the DAG presented in Figure 2.

Definition 17. The observables and the unknowns can be redefined as:

$$\begin{aligned}
P_Z(t, c) &= [P(T = t|Z = z_1, C = c), ..., \\
&\qquad P(T = t|Z = z_{N_Z}, C = c)]^T \\
P_A(c) &= [P(A = a_1|C = c), ..., P(A = a_{N_A}|C = c)]^T \\
Q_Z(t, c) &= [E(Y|T = t, Z = z_1, C = c), ..., \\
&\qquad E(Y|T = t, Z = z_{N_Z}, C = c)]^T \odot P_Z(t, c) \\
Q_A(t, c) &= [E(Y|T = t, A = a_1, C = c), ..., \\
&\qquad E(Y|T = t, A = a_{N_A}, C = c)]^T \odot P_A(c)
\end{aligned}$$

Proposition 4. *If for two different treatments t and t', there exists a vector b, such that*

$$bK_t = bK_{t'} = 0,$$

then the LATE can be estimated as:

$$E(Y(t) - Y(t')|w \in \Sigma(b)) = \sum_c P(C = c) \left(\frac{bB_t^+ Q_Z(t, c)}{bB_t^+ P_Z(t, c)} - \frac{bB_{t'}^+ Q_Z(t', c)}{bB_{t'}^+ P_Z(t', c)} \right), \tag{26}$$

where the sum should be replaced with an integral for continuous C. If it can additionally be assumed that $A \perp\!\!\!\perp C$, then Equation 26 can be simplified to:

$$E(Y(t) - Y(t')|w \in \Sigma(b)) = \frac{\sum_c P(C = c) bB_t^+ Q_Z(t, c)}{bB_t^+ P_Z(t)} - \frac{\sum_c P(C = c) bB_{t'}^+ Q_Z(t', c)}{bB_{t'}^+ P_Z(t')}.$$

Proof. From IC 4, following similar steps to the proof of Lemma 1, presented in Subsection A.3, we have:

$$E(Y(t)|w \in \Sigma(b_t), C = c) = \frac{\sum_{a \in \Pi(b_t)} E(Y|T = t, A = a, C = c) P(A = a|C = c)}{P(A \in \Pi(b_t)|C = c))}, \tag{27}$$

where positivity is guaranteed by the conditional version of the positivity assumption. Using Equation 27 and following

a similar proof to the one for Proposition 2, one can show that given IC 4-7:

$$P(T=t|Z=z,C=c)=\sum_a 1[T=t|A=a,Z=z]P(A=a|C=c),$$
$$E(Y|T=t,Z=z,C=c)P(T=t|Z=z,C=c)=\sum_a 1[T=t|A=a,Z=z]\,E(Y|T=t,A=a,C=c)P(A=a|C=c).$$

These equations can be reformulated as:

$$Q_Z(t,c)=B_tQ_A(t,c)$$
$$P_Z(t,c)=B_tP_A(c).$$

Therefore, similar to the proof of Theorem 1, presented in Subsection A.6, it can be shown that:

$$E(Y(t)|w\in\Sigma(b_t),C=c)=\frac{bB_t^+Q_Z(t,c)}{bB_t^+P_Z(t,c)}$$

Also, from IC 4 we have:

$$E(Y(t)|w\in\Sigma(b_t))=\sum_c E(Y(t)|w\in\Sigma(b_t),C=c)P(C=c),$$

where the sum should be replaced with an integral over the distribution of C for continuous C. Positivity in this equation is fulfilled by the assumption that all adherence sets are possible under all values of C.

Combining the last two equations yields:

$$E(Y(t)|w\in\Sigma(b_t))=\sum_c P(C=c)\frac{bB_t^+Q_Z(t,c)}{bB_t^+P_Z(t,c)}$$

For head-to-head comparisons, one needs to subtract the LATO of one treatment alternative from that of the other to obtain the result stated in the proposition.

If $A\perp\!\!\!\perp C$, we have:

$$P(A=a|C=c)=P(A=a)$$
$$P(A\in\Pi(b_t)|C=c)=P(A\in\Pi(b_t))$$

Equation 27 can then be rewritten as:

$$E(Y(t)|w\in\Sigma(b_t),C=c)=\frac{\sum_{a\in\Pi(b_t)}E(Y|T=t,A=a,C=c)P(A=a)}{P(A\in\Pi(b_t))}.$$

Following the same steps, one then arrives at the simplified expression given in the proposition.

Note that since T still is only a function of Z and A, B_ts, B_t^+s and K_ts remain unchanged here compared to when marginal independence can be assumed. Proposition 4 shows that if $A\perp\!\!\!\perp C$, it suffices to adjust for C in the model for Y. Otherwise, the CIV estimator should be integrated over the distribution of C. This might be computationally expensive. One can, however, construct a plug-in estimator that conserves consistency.

Corollary 2. *For continuous C, Corollary 1 also holds for the plug-in estimator:*

$$\hat{E}^{Adj}_{CIV}(Y(t)-Y(t')|w\in\Sigma(b))=\frac{bB_t^+\int_c\hat{Q}_Z(t,c)dp(c)}{bB_t^+\int_c\hat{P}_Z(t,c)dp(C)}-\frac{bB_{t'}^+\int_c\hat{Q}_Z(t',c)dp(c)}{bB_{t'}^+\int_c\hat{P}_Z(t',c)dp(c)},\tag{28}$$

Proof. Here, we want to prove:

$$E\left(\frac{bB_t^+\int_c\hat{Q}_Z(t,c)dp(c)}{bB_t^+\int_c\hat{P}_Z(t,c)dp(C)}\right)=E(Y(t)|w\in\Sigma(b)).$$

Denote variables Υ and Φ and function $f(\upsilon,\phi)$ as:

$$\Upsilon=bB_t^+\int_c\hat{Q}_Z(t,c)dp(c)$$
$$\Phi=bB_t^+\int_c\hat{P}_Z(t,c)dp(c)$$
$$f(\upsilon,\phi)=\frac{\upsilon}{\phi}.$$

Note that, similar to the proof of Corollary 1:

$$E(\hat{\Upsilon})=bB_t^+E(\int_c\hat{Q}_Z(t,c)dp(c))=bB_t^+Q_Z(t)$$
$$E(\hat{\Phi})=bB_t^+E(\int_c\hat{P}_Z(t,c)dp(c))=bB_t^+P_Z(t).$$

Following the same steps, one can then show:

$$E(f(\hat{\Upsilon},\hat{\Phi}))=f(\Upsilon,\Phi)+O(Var(\hat{\Phi}))+O(Cov(\hat{\Upsilon},\hat{\Phi})).$$

From the law of large numbers and homogeneity of n, we have:

$$O(Var(\hat{\Phi}))=O(Cov(\hat{\Upsilon},\hat{\Phi}))=O(n^{-1}).$$

Therefore:

$$E(f(\hat{\Upsilon},\hat{\Phi}))=f(\Upsilon,\Phi)+O(n^{-1})$$

and the proof is completed.

The natural way to obtain the plug-in estimates of the integrals in Corollary 2 is using standard models and marginalizing over C. Natural models for $P_z(t,c)$ are multinomial logistic or probit, while
$E(Y|T=t,Z=z,C=c)$ can be estimated by a (generalized) linear model (e.g. logistic regression if the outcome is binary). Corollary 2 states that as in the standard case, the plug-in estimator is asymptotically unbiased.

In a similar way, $L_Z(t,C)$ and $L_A(C)$ can be constructed by adjusting $L_Z(t)$ and L_A for C to obtain a modified version of Equation 29 under $X\perp\!\!\!\perp Z|C$ and $X\perp\!\!\!\perp T|A,C$.

B.7 Exchangeable Pseudo-Populations

Another way of thinking about the results presented in Subsection 3.7 is in terms of exchangeable pseudo-populations. Even though the framework presented in this paper is a non-parametric one, we can make a bridge to parametric modeling by creating a corresponding pseudo-population.

Corollary 3. *For a pair of treatments t and t', where b is a common solution to Equation 10, give to each observation w the following weight:*

$$W_w = \frac{P(T = t_w)}{P(Z = z_w)} \cdot \frac{bB^+_{t_w}[:, z_w]}{bB^+_{t_w} P_Z(t_w)},$$

where $B^+_{t_w}[:, z_w]$ is the column in $B^+_{t_w}$ corresponding to z_w. Then it can be shown that:

$$E(Y(t) - Y(t')|w \in \Sigma(b)) = \\ E(Y \cdot W|T = t) - E(Y \cdot W|T = t').$$

In other words, in the pseudo-population created by weighing each observation according to the above expression, $\{w|T = t\}$ and $\{w|T = t'\}$ are exchangeable. Given additional covariates X, under assumptions posed in Proposition 3, we have:

$$E(X \cdot W|T = t) = E(X \cdot W|T = t'). \tag{29}$$

Proof. According to Theorem 1, we have:

$$E(Y(t)|w \in \Sigma(b)) = \frac{bB^+_t Q_Z(t)}{bB^+_t P_Z(t)}.$$

Now, we are going to show:

$$E(Y \cdot W|T = t) = \frac{bB^+_t Q_Z(t)}{bB^+_t P_Z(t)}. \tag{30}$$

Note that W is only a function of T and Z:

$$W(t_w, z_w) = \frac{P(T = t_w)}{P(Z = z_w)} \cdot \frac{bB^+_{t_w}[:, z_w]}{bB^+_{t_w} P_Z(t_w)}$$

Starting from the left-hand side of Equation 30, we have:

$$\begin{aligned} E(Y \cdot W|T = t) &= \sum_z P(Z = z)E(Y \cdot W|T = t, Z = z) \\ &= \sum_z P(Z = z)W(t, z)E(Y|T = t, Z = z) \\ &= \sum_z \frac{P(Z = z)W(t, z)E(Y \cdot 1[T = t]|Z = z)}{P(T = t)} \\ &= \sum_z \frac{bB^+_t[:, z]E(Y \cdot 1[T = t]|Z = z)}{bB^+_t P_Z(t)} \\ &= \frac{\sum_z bB^+_t[:, z]E(Y \cdot 1[T = t]|Z = z)}{bB^+_t P_Z(t)} \\ &= \frac{bB^+_t Q_Z(t)}{bB^+_t P_Z(t)}, \end{aligned}$$

where in the second line, W can be taken out of the expectation because given T and Z, W is constant. Replacing Y with X and following the same steps yields Equation 29.

B.8 Estimators in the Simulation Study

The following is the mathematical representation of the estimators considered in the simulation study:

$$\hat{E}_{\text{Naive}}(Y(t_i) - Y(t_j)) = \hat{E}(Y|T = t_i) - \hat{E}(Y|T = t_j)$$

$$\hat{E}_{\text{CIV}}(Y(t_i) - Y(t')) = \frac{bB^+_{t_i}\hat{Q}_Z(t_i)}{bB^+_{t_i}\hat{P}_Z(t_i)} - \frac{bB^+_{t_j}\hat{Q}_Z(t_j)}{bB^+_{t_j}\hat{P}_Z(t_j)}$$

$$\hat{E}_{\text{DIV}}(Y(t_i) - Y(t_j)) = \\ \frac{\hat{E}(Y|Z \in \zeta_i, T \in \{t_i, t_j\}) - \hat{E}(Y|Z \in \zeta_j, T \in \{t_i, t_j\})}{\hat{P}(T = t_i|Z \in \zeta_i, T \in \{t_i, t_j\}) - \hat{P}(T = t_j|Z \in \zeta_j, T \in \{t_i, t_j\})},$$

where $\zeta_i = \{z_j = (t'_1, t'_2, t'_3)|t'_k = t_i, t'_l = t_j, k < l\}$
and $\zeta_j = \{z_j = (t'_1, t'_2, t'_3)|t'_k = t_i, t'_l = t_j, k > l\}$

B.9 Target Trial Emulation

Our approach fits nicely into the concept of target trial emulations[23]. The concept of adherence sets can be seen as emulating a trial with treatment choices restricted to the choices in the adherence set. E.g. the result comparing certulizumab and etanercept is defined in adherence sets which excludes infliximab. The emulated trial could then be a platform trial where the participant and the investigator prior to randomization decide which treatments are available and relevant for the patient. A busy participant would then not select infliximab as relevant because she/he would need to come in to the hospital to have an infusion. The patient would then be randomized to one of the other alternatives. Note also our estimator would estimates a treatment policy strategy estimand since we do not consider any intercurrent events after the treatment choice decision.

B.10 Relevance in the Clinical Case

A strong causal effect by the instrument on the treatment is necessary for IV estimates to be trustworthy. In Figure 4, adherence to the NDPC recommendations appears to be generally high. The exception is in the 2010 period. This could potentially be problematic for our method, since one of the underlying assumptions is $Z \perp\!\!\!\perp A$. One could speculate that in 2010, NDPC was still a relatively new arrangement and physicians were skeptical of choosing treatments based on an economically motivated recommendation. If this was true, the assumption $Z \perp\!\!\!\perp A$ would be violated. One could therefore consider removing decision teams starting treatment in 2010 from the data set. However, the distribution of decision teams choosing the different alternatives in 2010 could also be explained clinically. Infliximab is only applied through intravenous injection by health personnel at a hospital, which means patients must visit the hospital regularly to receive the treatment. The other treatments can also be applied through subcutaneous injection at home. Infliximab might therefore be unpopular in cases where the patient cannot easily visit the hospital. Also, golimumab, which was the second cheapest option in 2010, is not recommended for pregnant women due to limited evidence supporting its safety in this group. The relative unpopularity of these two treatment alternatives in 2010 might therefore simply be explained by random variations in the patient population from year to year. A clinical explanation similar to this would not violate any of the assumptions underlying

our method. In our analysis we will therefore include data from all available years.

B.11 Treatment Distribution in the Simulated Data

Comparing the histograms presented in Figure 4 and 6, one can argue that the treatment probability distribution in the simulated data sets resembles the observed treatment probability distribution in the clinical use-case. In the simulated data, the probability of t_3 peaks at around 0.3 even when it is the most encouraged alternative under $Z = z_4$. One can then say that t_3 represents an unpopular alternative similar to infliximab in the real-world case. Similarly, t_1 and t_2 are chosen by almost 0.4 of the decision teams even when they are not the most encouraged alternatives under $Z = z_2$ and $Z = z_1$, respectively. These treatments represent popular alternatives like certolizumab and etanercept.

B.12 Handling of Missing Data

As our intention in this paper is only to illustrate the applicability of our method in a clinical use-case, we have chosen a simple method for handling missing data. Initially, 984 patient records were considered eligible (initiated treatment with one of the five TNF inhibitors between 2010 and 2019). Of these, $50(5.1\%)$ patients had missing outcome and were excluded from the analysis population assuming they are missing completely at random. Of the 934 in the analysis population, $216(23.1\%)$ had their treatment course terminated earlier than 3 months after the start of the treatment. For these patients, the outcome was set to non-remission, making the outcome a composite endpoint of remission and tolerability. Another 9 (1%) patients continued their treatment passed the 3-months mark but failed to measure CRP at that visit. Erythrocyte sedimentation rate (ESR) was then used to calculate DAS28 ESR[21] and derive clinical remission.

B.13 Adjustment for Baseline

Figure 7 shows the remission rate against calendar time. One can see that the average remission rate increases almost linearly from under 0.3 to above 0.5 from 2010 to 2019. This might be explained by improving clinical practices or a change in the target population towards milder disease at baseline. At the same time, one can see that adalimumab was one of the most expensive treatments in the periods $2010 - 2018$, while it was the cheapest treatment alternative in 2019. This can be explained by the emergence of biosimilar (competitor) drugs on the market. While it is implausible that the price change itself could affect the remission rate of RA patients other than through the increase in adalimumab use, this co-variation could lead to imprecise estimates of the adalimumab efficacy given the limited number of realizations of the instrument.

As a sensitivity analysis, we will adjust our estimates for DAS28 CRP at baseline. We denote DAS28 CRP at baseline as C and assume $T \perp\!\!\!\perp C|A, Z$. Then we have:

$$
\begin{aligned}
&Q_Z(t,c)[z] = \\
&\qquad E(Y|T=t, Z=z, C=c)P(T=t|Z=z, C=c).
\end{aligned}
$$

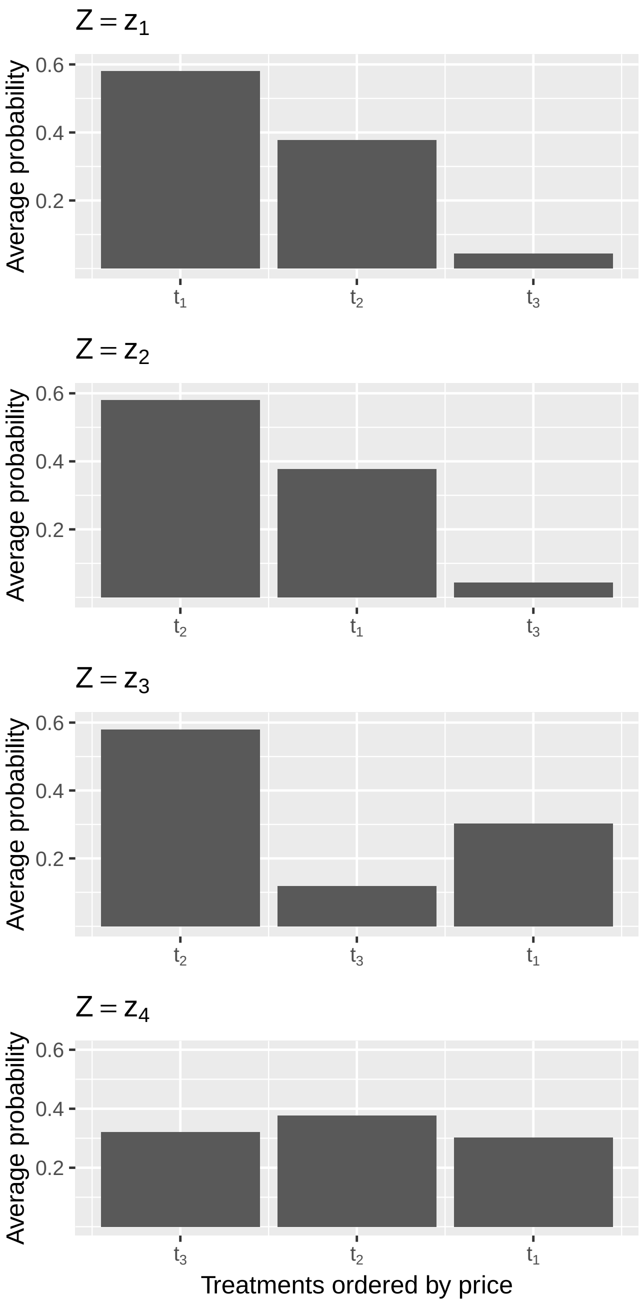

Figure 6. Average probability of each treatment over all simulated data sets under each value of the instrument.

The terms in this equation can be modeled with an ordinary and a multinomial logistic regression model. We adjust for C linearly in both models and calculate marginal effects for all (t, z)-pairs. We then denote the marginal effects from the multinomial logistic regression model as $\hat{P}_Z^{\text{GLM}}(t)$ and the product of the marginal effects from the two models as $\hat{Q}_Z^{\text{GLM}}(t)$. Then, under some regularity conditions, one can show:

$$
\begin{aligned}
\int_c bB_t^+ \hat{Q}_Z(t,c)dp(c) &= bB_t^+ \int_c \hat{Q}_Z(t,c)dp(c) \\
&= bB_t^+ \hat{Q}_Z^{\text{GLM}}(t) \\
\int_c bB_t^+ \hat{P}_Z(t,c)dp(c) &= bB_t^+ \int_c \hat{P}_Z(t,c)dp(c) \\
&= bB_t^+ \hat{P}_Z^{\text{GLM}}(t).
\end{aligned}
$$

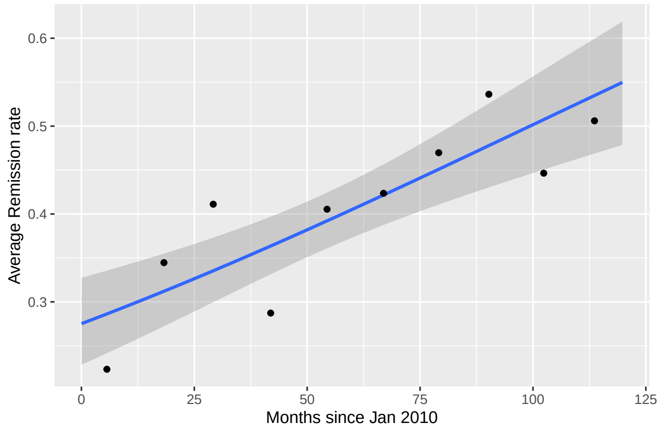

Figure 7. Average remission rate under each NDPC period. The line represents the pooled logistic regression fit to the data from these periods.

These expressions can then be plugged into Equation 28 to obtain estimates for the LATEs. The results are shown in Table 5.

Comparing the results presented in Tables 4 and 5, one can see that all crude CIs overlap to a large extent with their corresponding adjusted CIs. We therefore conclude that our estimates are not sensitive to adjustment for baseline DAS28 CRP. On the other hand, the adjusted CIs are not narrower than the crude CIs. One could therefore claim that this adjustment did not improve efficiency. Given that the crude estimates were obtained non-parametrically while logistic regression was used to obtain the adjusted estimates, the crude results are to be preferred.

LATE	$\hat{E}^{\mathrm{A}}(.)$	Adjusted CI	n^{A}
$E(Y(\mathrm{Inf}) - Y(\mathrm{Cer}) \mid \{\mathrm{Inf}, \mathrm{Cer}\} \subseteq A)$	0.40	$(0.04, 0.80)$	2485
$E(Y(\mathrm{Inf}) - Y(\mathrm{Eta}) \mid \{\mathrm{Inf}, \mathrm{Eta}\} \subseteq A)$	0.34	$(-0.20, 0.91)$	2311
$E(Y(\mathrm{Ada}) - Y(\mathrm{Eta}) \mid A = \{\mathrm{Ada}, \mathrm{Eta}\})$	-0.05	$(-0.91, 0.89)$	1497
$E(Y(\mathrm{Ada}) - Y(\mathrm{Gol}) \mid A = \{\mathrm{Ada}, \mathrm{Gol}\})$	-0.38	$(-0.72, -0.05)$	2500
$E(Y(\mathrm{Cer}) - Y(\mathrm{Eta}) \mid \{\mathrm{Cer}, \mathrm{Eta}\} \subseteq A \subseteq \{\mathrm{Ada}, \mathrm{Cer}, \mathrm{Eta}, \mathrm{Gol}\})$	0.07	$(-0.11, 0.25)$	2500
$E(Y(\mathrm{Cer}) - Y(\mathrm{Gol}) \mid \{\mathrm{Cer}, \mathrm{Gol}\} \subseteq A \subseteq \{\mathrm{Ada}, \mathrm{Cer}, \mathrm{Gol}\})$	-0.38	$(-0.94, 0.44)$	1856
$E(Y(\mathrm{Cer}) - Y(\mathrm{Gol}) \mid \{\mathrm{Cer}, \mathrm{Gol}\} \subseteq A \subseteq \{\mathrm{Ada}, \mathrm{Cer}, \mathrm{Eta}, \mathrm{Gol}\})$	0.25	$(0.03, 0.49)$	2500
$E(Y(\mathrm{Cer}) - Y(\mathrm{Gol}) \mid \{\mathrm{Cer}, \mathrm{Gol}\} \subseteq A \subseteq \{\mathrm{Cer}, \mathrm{Eta}, \mathrm{Gol}\})$	0.30	$(0.06, 0.55)$	2500
$E(Y(\mathrm{Eta}) - Y(\mathrm{Gol}) \mid \{\mathrm{Eta}, \mathrm{Gol}\} \subseteq A \subseteq \{\mathrm{Ada}, \mathrm{Eta}, \mathrm{Gol}\})$	0.24	$(-0.90, 0.96)$	744

Table 5. The first column lists all identifiable LATEs specifying their corresponding sub-population. Adjusted LATE estimates are given under $\hat{E}^{\mathrm{A}}(.)$. Adjusted CI are the 95% bootstrapping confidence interval for these estimates. The number of replications that yielded an adjusted estimate in the ± 1 interval for each LATE are reported as n^{A}. Inf=Infliximab; Gol = Golimumab; Cer = Certolizumab pegol; Eta = Etanercept; Ada = Adalimumab
